# Contraceptive use, unmet need, and demand satisfied for family planning across Cameroon: a subnational study including indirect effects of COVID-19 and armed conflict on projections

**DOI:** 10.1186/s44263-024-00071-4

**Published:** 2024-07-03

**Authors:** Raïssa Shiyghan Nsashiyi, Md Mizanur Rahman, Lawrence Monah Ndam, Masahiro Hashizume

**Affiliations:** 1 Institute for Nature, Health, and Agricultural Research (INHAR), P.O Box 71, Buea, Southwest Region Cameroon; 2 Hitotsubashi Institute for Advanced Study, University of Hitotsubashi, 2-1 Naka, Kunitachi, Tokyo, 186-8601 Japan; 3 Department of Agronomic and Applied Molecular Sciences, Faculty of Agriculture and Veterinary Medicine, University of Buea, P.O Box 63, Buea, Southwest Region Cameroon; 4 Department of Global Health Policy, Graduate School of Medicine, The University of Tokyo, 7-3-1, Hongo, Bunkyo-ku, Tokyo, Japan

**Keywords:** Family planning, Contraceptive, Unmet need, Demand satisfied, Services coverage, COVID-19, Armed conflict

## Abstract

**Background:**

Cameroon is among countries that have made notable investments nationwide in line with the Family Planning 2030 initiative. This study examines the progress made across the 10 regions and 58 divisions of the country, including potential impairments following COVID-19 and armed conflict.

**Methods:**

In this time-series analysis, parameters were sourced from 5 Demographic and Health Surveys and 3 Multiple Indicator Cluster Surveys conducted between 1991 and 2018. The Family Planning Estimation Tool (FPET) was used to estimate key family planning indicators among married women of reproductive age. Data from official country reports and FPET results were incorporated into Bayesian models to assess how projections (from 2020 to 2030) would vary given varying contractions (i.e., 0%, 5%, 10%, and 25% annually) to services coverage.

**Results:**

Nationally, modern contraceptive prevalence rate (mCPR) and demand satisfied with modern (contraceptive) methods reached 16.8% (95% credible interval 12.0 to 23.0) and 37.6% (28.7 to 47.0), respectively; and unmet need for modern methods decreased to 27.9% (22.9 to 33.7) in 2022. Notable improvements were observed in the *East* region and *Boumba et Ngoko* division, with annual mCPR trends of 2.1 percentage points (%p) (− 0.2 to 4.2) and 7.0%p (4.5 to 9.3) from 2015–2020; and 2030 projections of 58.7% (41.3 to 74.0) and 79.1% (65.0 to 89.0), respectively. The least performing in terms of demand satisfied with modern methods include *Adamawa* at the regional and *Boyo* at the divisional level, with 2030 projections of 45.5% (26.5 to 65.1) and 0.4% (0.2 to 0.8), respectively. The northern regions (*Adamawa*, *Far North*, and *North*) recorded the lowest levels of unmet need for modern methods. To achieve ≥ 75% demand satisfied with modern methods in 2030, an additional 1.4 (0.6 to 2.2) million modern contraceptive users are required. Given large reductions (− 25% annually) in services coverage post-COVID-19/armed conflict (2020 to 2030), the *South* region could experience the most significant contraction in projected mCPR, with a decrease of − 9.2%.

**Conclusions:**

Family planning outcomes vary significantly across subnational territories of Cameroon. While the *East* region shows notable success, greater attention is needed in the northern regions. Strategies must be adaptive to address unprecedented emergencies that may disrupt access to services.

**Supplementary Information:**

The online version contains supplementary material available at 10.1186/s44263-024-00071-4.

## Background

Family planning, particularly contraceptive-assisted birth spacing and limiting, is key among reproductive health interventions linked to the health, survival and well-being of women and children worldwide [1]. In 2012, assessments revealed that contraceptives avert around 230 million unintended pregnancies and 272 thousand maternal deaths annually [2]. Therefore, global efforts including the Sustainable Development Goals–SDG 3.7 to ensure universal access to sexual and reproductive healthcare services [3] have been geared toward improving access to basic family planning care, particularly in resource-limited settings [1]. Such efforts are crucial in the face of emerging challenges, such as the coronavirus disease 2019 (COVID-19) pandemic, which can disrupt access to and utilization of family planning services [4].

The Family Planning 2030 (FP2030) is a global initiative that was first launched in 2012 as Family Planning 2020 (FP2020), to enhance access to modern contraceptives for 120 million additional women and adolescent girls in 69 low- and middle-income countries by 2020 [5]. Cameroon is one among these countries that made substantial investments toward FP2020/FP2030, including increases in the budget line for the procurement of contraceptive commodities, training family planning personnel, and expanding services nationwide [6]. Pre-FP2020 analyses suggested that modern contraceptive use needed to increase on average by at least 1.4 percentage points (%p) annually in FP2020 countries to achieve the set targets [1]. Regarding the sustainable development goals (SDGs), a 2030 target of ≥ 75% demand satisfied with modern contraceptive methods has been proposed for attainment by all countries [7]. With such initiatives, demands heightened for comparative assessments that would reveal the gains made and remaining gaps, such that would inform future endeavors [8, 9].

To date, however, emphasis has been on assessments of family planning in larger population groups. With support from the Gates Foundation, Avenir Health (a global health organization) implements the Track20 Project to monitor progress towards achieving the goals of the FP2020/FP2030 initiative [10]. The Track20 Project works with governments of FP2020/FP2030 focus countries to collect data on family planning. The data are then analyzed using the Family Planning Estimation Tool (FPET) [11] to derive country-level estimates of family planning indicators, such as the modern contraceptive prevalence rate and unmet need for family planning [12, 13]. Still, given how within-country disparities could hamper overall progress and the emphasis on equity in the SDG era [3], assessments of smaller population groups have become crucial.

While a few subnational assessments have been conducted [8, 9], those that cover second-level administrative units have been scarcely published in academic journals. Subnational estimations are necessary in Cameroon as none had been previously conducted. Recent reports show that with an estimated modern contraceptive prevalence rate of 21.8% [95% credible interval (CrI) 13.2 to 29.7], the country is lagging behind its FP2030 counterparts in East and Southern Africa by at least 17% and 40%, respectively [12]. A smaller-area assessment is necessary to monitor whether progress in each of the administrative units could contribute to attaining set targets (e.g., SDG and FP2020/FP2030) at the national level. Importantly, Track20 provides documentation for preparing files and running the FPET at the subnational level [13]. For Cameroon, a subnational analysis would be crucial to inform family planning policies at the local levels where programme implementation mainly occurs [14].

Furthermore, in the face of unprecedented threats [15], such as infectious disease outbreaks like the most recent COVID-19 pandemic, which hinder routine health services, current studies need to consider how these disruptions could adversely impact reproductive or family planning health. Evidence from low- and middle-income countries suggests negative consequences to health that are linked to disruptions in health systems encountered during COVID-19 [15, 16] or following armed conflicts [17]. In the case of Cameroon, both the outbreak of COVID-19 in 2020 and the ongoing armed conflict that began in 2016 have severely strained the provision of routine healthcare [18, 19], including family planning services. Therefore, this study aims to assess levels, trends, and projections of family planning across Cameroon’s first- (10 regions) and second- (58 divisions) level administrative units. Estimates include modern contraceptive use, unmet need for modern methods, and demand satisfied with modern methods. Additionally, the study hypothetically examines the indirect effects of health services coverage reductions due to COVID-19/armed conflict on family planning projections between 2020 and 2030.

## Methods

### Setting

Cameroon is a lower-middle-income country located between West and Central Africa, with a population of about 27 million [20]. The health sector in Cameroon is organized into three levels, mostly corresponding with the country’s administrative units [14]. These include 10 first-level, 58 second-level, and 360 third-level administrative units, named regions, divisions, and sub-divisions, respectively [20]. Over the past few decades, Cameroon has made notable progress in healthcare, with a particular focus on family planning. From the early 90s to the present, the use of modern contraceptives for family planning among married women of reproductive age increased by more than 15% [21]. In 1994, a policy change by the government led to the expansion of family planning care, including the integration of the “Mother and Child Health Care/Family Planning” centers, into all levels of primary healthcare delivery [22]. Starting from 2014 onwards, substantial investments were made toward the FP2020 initiative in Cameroon. This included directing an additional yearly budget (≈ 350,000 US dollars for 2017) toward the procurement of family planning commodities, particularly the easy-to-administer Depot medroxyprogesterone acetate subcutaneous (DMPA-SC) injectable contraceptive recommended under the FP2020 initiative [6]. The Ministry of Public Health initiated community-based projects for the distribution of long-acting modern contraceptives, including implants [such as etonogestrel implant (Implanon NXT)] and injectables (such as DMPA-SC). Additionally, 690 family planning providers were trained, the “SMS for Life” software was introduced to update contraceptive stockpiles nationwide, and caravan campaigns were launched to provide free services and raise awareness about family planning [6].

### Data

We performed a time-series study employing several data sources specified below.

*For estimation of family planning indicators* (data inputted into the FPET) [11]: (i) 34 country-, 300 regional-, and 904 divisional-level observations of the various family planning indicators were sourced from cross-sectional surveys, including Demographic and Health Surveys (DHS) conducted in 1991 [23], 1998 [24], 2004 [25], 2011 [26], and 2018 [27], and Multiple Indicator Cluster Surveys (MICS) conducted in 2000 [28], 2006 [29], and 2014 [30]. At the level of the divisions, using “QGIS 3.8” (an open-source geographic information system software) [31], DHS data were reclassified according to the geolocation of clusters. More details regarding the survey data are presented in Additional File 1. (ii) Subnational estimates of married women (i.e., the base population) and unmarried women of reproductive age were derived via Bayesian hierarchical modelling, using census data from the Integrated Public Use Microdata Series International [32], DHS data [23–27], and World Population Prospects life tables [33] for Cameroon.

The population model to estimate counts of either married women or unmarried women of reproductive age for each division and region of Cameroon was based on standard regression forecasting approaches. These approaches assume that factors influencing population size have measured effects on population change over time [34, 35]. Key demographic drivers, including fertility rates, survival rates, and net-migration [36], were incorporated as covariates. Drawing from proposed Bayesian methodologies on small-area population forecasting [37–39], the model was built such that for each age, changes in the log-transformed population size will be influenced by patterns of age-specific fertility and survival rates, as well as net-migration proportion (for the corresponding ages) within each division, region, and year. The time-series approach allowed for capturing trends over time. The base model was followed by standard regression forecasting, which involved deriving out-of-sample population predictions for the years 1990 to 2030 based on the relationships established between the dependent and independent variables [40]. Best estimates were computed as the median of generated samples from the posterior distributions via the Markov Chain Monte Carlo algorithm. The Stata Statistical Software: Release 17.1 was used for this analysis. More details on the population estimation are provided in Additional File 1 and have been published elsewhere [41].

*For estimation of indirect effects of health services coverage reductions*: Regional-level (i) estimates of each family planning indicator, including demand for family planning, were derived from the FPET for the years 2020 to 2030, (ii) data for health services provision (composed of per capita health workers, facilities, and budget) and health services affordability (per poverty rate) were computed based on figures provided in official government reports on health and finance [14, 42, 43].

### Variables

Modern contraceptive prevalence rate (mCPR) measures the percentage of women who are currently using, or whose partner is currently using at least one modern method of contraception [44]. Unmet need for family planning shows the percentage of fecund women, who do not want any more children, are undecided, or want to delay the birth of the next child for at least 2 years, but are currently not using a modern method of contraception, and of pregnant or postpartum amenorrheic women who wanted to delay or did not want their current or last pregnancy. Demand for family planning satisfied with modern methods is an indicator of the percentage of modern contraceptive use among women who have a demand for family planning. All estimates are for women of reproductive age, i.e., 15 to 49 years, who are married or in a union.

### Statistical analysis

#### The family planning estimation model

Estimates were derived using the FPET [11]. The FPET includes a global family planning estimation model (FPEM) that merges country-specific systematic trends in total contraceptive prevalence, the ratio of modern to total prevalence, and the ratio of unmet need to no contraceptive use, within a Bayesian hierarchical model. The parameters are modeled based on logistic growth curves with a time-series model that captures fluctuations around the trends [45]. These include contraceptive prevalence, unmet need for family planning, and associated parameters. The model accounts for differences in inputted data sources, population samples, and contraceptive methods (modern or traditional) included in the measure [12]. Country-level model specifications include underlying contraceptive trend assumptions of a gradual increase at the early or low prevalence phase, more rapid increases in the intermediate phase, and a slowdown at the late phase when high prevalence is achieved. Unmet need is derived following a statistically defined relation between total contraceptive prevalence and unmet need. Importantly, the FPEM’s hierarchical set-up allows for country-specific changes to be influenced by sub-regional, regional, and global rates/trends [12, 45].

The subnational-FPEM, an extension of the global-FPEM, follows the same setup [8, 46]. However, for each sub-population parameter, rates, trends, and data above the country level are rather fixed-point estimates from the most recent global model run. This model includes extra level(s) to the hierarchy for subnational data, thus, changing from a subregion-country to a country-subpopulation set-up, with priors informed by the global model. Also, given data uncertainty and variability for smaller geographical areas, spatial models have been incorporated that allow for greater pooling of information. Estimates include the median and 95% CrI, representing the 50.0th, and the 2.5th and 97.5th percentiles of posterior distributions, respectively.

#### Assessment of progress in family planning

Annual changes in mCPR were computed for 5 years post- (2015 to 2020) Cameroon’s first FP2020 investments [6]. Reference is made to the recommended 1.4%p increases under the FP2020 initiative [1]. The assessment also includes computations of what increase in terms of percentage of and how many additional users of modern contraceptives are required to attain the proposed target of ≥ 75% demand satisfied with modern methods in 2030 [7]. Similar to New et al*.* (2017) [8], the increase in percentage of and additional users of modern contraceptives required to meet the ≥ 75% target were respectively computed by (i) subtracting the percentage of women who were using modern contraceptives in 2022 from 75% of the projected percentage total demand in 2030, and (ii) subtracting the number of women who were using modern contraceptives in 2022 from 75% of the projected total demand in 2030, respectively.

#### Modelling indirect effects of health services coverage reductions on family planning projections

Similar to Roberton et al. (2020), a simple health systems framework [15] (see Additional File 2: Fig. S1) was adapted to develop assumptions for likely changes in the levels of family planning, which could result depending on how severely the COVID-19 pandemic and/or armed conflict affect the coverage of health services in Cameroon. The framework assumes that family planning prevalence is dependent on the coverage of relevant services, which is composed of provision and utilization. Provision is a product of services supplies, including human, material, and financial resources, while utilization is a product of services demand and affordability [15]. Similar to earlier assessments [15, 47], linear assumptions were made regarding the effectiveness of health services coverage (a proxy for family planning services coverage) on projections of family planning. Incorporating the aforementioned components, different scenarios were created to represent possible real-world reductions in services coverage. Changes in either provision or utilization, each ranging from 0%, − 5%, − 10%, to − 25% yearly, were computed for the years 2020 to 2030. Likewise drawing from Roberton et al. (2020), these % reductions were relatively defined as none (0%), small (5%), moderate (10%), and large (25%) [15], respectively, per annum. Equations;

1 $$\text{Change in FP indicator}=\text{Change in Coverage of Services}$$

2 $$\text{Change in FP indicator}=\text{Change in }(\text{Provision}\times \text{Utilization})$$

3 $$\text{Change in FP indicator}=[(\text{Provision})(1-x)\times (\text{Utilization})(1-y)]$$

4 $$\text{Change in FP indicator }= [(\text{HRH}\,*\,\text{FH}\,*\,\text{HB})(1-x) ]\times [(\text{Demand}\,*\,\text{Affordability})(1-y)]$$

5 $$\text{Change in FP indicator }= [(\text{HRH}\,*\,\text{FH}\,*\,\text{HB})\,(1-x) ] \times [(\text{Demand}\,*\,[1-\text{Poverty rate}])\,(1-y)]$$

where $mFP$ = family planning indicator. Provision is composed of per capita human resources, facilities, and budget for health, represented as $\text{HRH}$, $\text{FH}$, and $\text{HB}$, respectively. Components of utilization; (i) Demand is defined as the change in demand for family planning (based on estimates from the FPET), (ii) *Affordability* = 1 – *Poverty rate*, where, poverty rate equals the percentage of the population living below the poverty line [48]. Lastly, $x$ and $y$ equal the reduction rates for provision and utilization, respectively. This analysis was conducted using the Stata Statistical Software: Release 17.1. Further specifications of the model are provided in Additional File 2.

Though simplistic, the formula provides a structure to develop hypothetical scenarios for examining possible swings in the course of projections. It is important to note that the formula captures only the quantitative dimensions of services provision, not the qualitative aspects. Here, only mCPR was modeled as it is the only indicator that showed statically significant associations (*p* < 0.05) with both predictors, namely provision and utilization (Additional File 2: Table S4).

## Results

### Levels of family planning

**Fig. 1:**
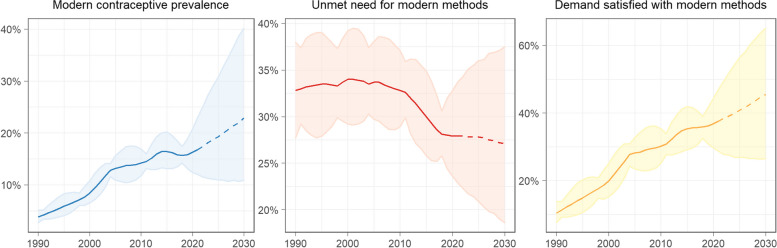
Estimates and projections of use, unmet need, and demand satisfied for modern methods in Cameroon Lines represent the median and shaded areas represent 95% credible intervals. Modern methods = modern contraceptive methods

Figure 1 illustrates country-level estimates of mCPR, unmet need for, and demand satisfied with modern methods from 1990 to 2030. Overall, there have been consistent upward trends in mCPR and demand satisfied with modern methods, reaching 16.8% (95% CrI 12.0 to 23.0) and 37.6% (28.7 to 47.0), respectively; and steady decreases in the unmet need for modern methods to 27.9% (22.9 to 33.7) in 2022.

Table 1 shows country-, regional-, and divisional-level estimates of mCPR, unmet need for and demand satisfied with modern methods in 2015 and projections for 2030. Each indicator shows large variations across the administrative units. At the regional level in 2015, the highest estimates of mCPR and demand satisfied with modern methods were recorded in the *Center* region, at 26.1% (21.6 to 31.2) and 44.7% (37.8 to 51.4), respectively; and the lowest estimates of unmet need for modern methods in the *Far North* region, at 23.1% (19.5 to 27.2) (Table 1). Regarding projections for 2030, the highest estimates of mCPR and demand satisfied with modern methods are expected in the *East* region, at 38.4% (21.5 to 57.3) and 61.4% (39.8 to 79.1), respectively, and the lowest levels of unmet need for modern methods in the *North* region, at 22.2% (15.6 to 30.8). The least performing regions in terms of both mCPR and demand satisfied with modern methods will include the *Adamawa*, *Far North*, and *North*. The respective 2030 projections in these regions are 22.9% (10.9 to 40.2), 10.8% (4.8 to 22.5), and 10.8% (4.9 to 22.6) for mCPR, and 45.5% (26.5 to 65.1), 31.8% (16.5 to 52.2), and 32.7% (17.0 to 52.9) for demand satisfied with modern methods. In terms of unmet need for modern methods, the least performing regions in 2030 will include the *South*, *Littoral*, and *Southwest*, with the highest estimated projections of 29.7% (21.3 to 39.9), 28.7% (19.8 to 40.3), and 28.2% (19.5 to 38.9), respectively.

**Table 1:** Modern contraceptives use, unmet need, demand satisfied, and attainability of 75% demand satisfied across Cameroon

	**Modern contraceptive methods**						**75% demand satisfied with modern methods (2030)**		
**Country** **Region** **Division**	**Use of (2015)**	**Use of (2030)**	**Unmet need for (2015)**	**Unmet need for (2030)**	**Demand satisfied with (2015)**	**Demand satisfied with (2030)**	**Attainment probability**	**Required increase in users of modern methods (compared with 2022)**	
								**Percentage (%)**	**Numbers (‘000)**
**CAMEROON**	**16.4 (13.1–20.2)**	**22.9 (10.9–40.2)**	**30.0 (25.8–34.9)**	**27.1 (18.6–37.5)**	**35.3 (29.1–41.9)**	**45.5 (26.5–65.1)**	**..**	**21.4 (4.0–37.4)**	**1429.7 (569.7–2243.2)**
**Adamawa**	**8.5 (6.7–10.5)**	**9.4 (4.0–20.1)**	**26.9 (23.2–31.3)**	**26.8 (19.0–36.6)**	**23.9 (19.2–28.9)**	**25.9 (12.6–44.8)**	**..**	**21.0 (9.9–32.9)**	**63.0 (33.8–95.0)**
**Djerem**	9.7 (7.6–12.4)	17.0 (8.1–31.9)	26.3 (22.4–30.6)	30.1 (21.5–40.1)	27.0 (21.6–33.0)	36.0 (19.8–55.6)	..	23.4 (8.8**–**37.5)	6.0 (2.6**–**9.4)
**Faro et Déo**	1.5 (1.1–2.1)	0.2 (0.1–0.5)	21.2 (17.6–25.1)	23.9 (16.2–33.6)	6.5 (4.6–9.1)	0.8 (0.3–2.2)	..	18.0 (12.1**–**25.3)	2.3 (1.6**–**3.3)
**Mayo Banyo**	3.3 (2.5–4.3)	3.5 (1.5–8.0)	20.7 (17.1–24.6)	23.6 (16.0–33.0)	13.7 (10.5–17.8)	13.0 (5.5–26.6)	..	17.9 (10.6**–**26.4)	9.2 (5.6**–**13.3)
**Mbéré**	3.3 (2.5–4.3)	4.8 (2.1–11.2)	28.3 (24.1–32.8)	25.3 (17.8–34.9)	10.4 (7.9–13.7)	16.0 (6.9–31.5)	..	19.6 (11.6**–**29.3)	9.7 (5.9**–**14.3)
**Vina**	10.4 (8.0–13.3)	12.6 (5.5–25.0)	28.3 (23.9–32.9)	29.6 (21.1–39.7)	26.8 (21.4–33.3)	30.0 (14.9–49.0)	..	22.9 (10.1**–**35.8)	37.2 (20.3**–**54.7)
**Centre**	**26.1 (21.6–31.2)**	**33.7 (17.7–52.7)**	**32.3 (27.9–37.1)**	**25.2 (16.3–34.6)**	**44.7 (37.8–51.4)**	**56.8 (36.9–75.3)**	**3.0**	**17.9 (**− **1.7–34.9)**	**358.4 (143.1–545.4)**
**Haute Sanaga**	17.6 (14.1–21.7)	22.9 (11.4–40.6)	41.5 (36.9–46.7)	33.5 (23.8–44.3)	29.7 (24.3–35.6)	40.4 (23.2–61.2)	..	26.1 (9.7**–**40.6)	3.7 (1.5**–**5.7)
**Lekié**	25.5 (20.9–30.8)	33.0 (17.7–51.9)	29.7 (25.8–34.1)	22.8 (14.7–31.6)	46.2 (39.3–53.0)	58.8 (39.1–76.8)	4.0	15.7 (− 3.1**–**32.4)	5.1 (− 1.1**–**10.5)
**Mbam et Inoubou**	30.7 (25.5–36.5)	40.2 (22.9–59.8)	28.2 (24.1–32.7)	19.7 (11.6–29.0)	52.2 (45.1–59.1)	66.9 (46.2–83.3)	18.0	12.2 (− 7.5**–**29.9)	4.3 (− 0.8**–**8.8)
**Mbam et Kim**	22.4 (18.0–27.8)	35.1 (19.2–53.7)	30.3 (26.0–35.2)	24.4 (16.0–34.6)	42.5 (35.3–50.1)	58.6 (38.6–76.0)	3.0	17.6 (− 2.1**–**34.8)	2.7 (− 0.3**–**5.2)
**Mefou et Afamba**	31.6 (26.1–37.7)	37.2 (21.0–56.8)	28.2 (24.1–32.7)	24.7 (15.9–34.8)	52.8 (45.8–60.3)	59.8 (40.0–77.6)	5.0	16.4 (− 3.1**–**33.2)	4.1 (0.5**–**7.2)
**Mefou et Akono**	28.1 (22.9–33.6)	33.5 (18.0–53.7)	30.4 (26.2–35.1)	23.3 (14.4–33.5)	48.0 (40.9–54.9)	58.6 (38.0–78.0)	5.0	16.2 (− 3.2**–**33.2)	0.6 (− 0.3**–**1.3)
**Mfoundi**	25.2 (20.6–29.8)	33.0 (17.9–51.2)	32.1 (27.5–36.9)	25.5 (17.0–35.5)	43.9 (37.3–50.7)	56.1 (36.6–73.8)	2.0	18.1 (− 0.7**–**34.4)	320.3 (148.0**–**471.3)
**Nyong et Kéllé**	22.5 (18.1–27.5)	25.2 (12.7–43.3)	48.4 (43.6–53.4)	40.2 (29.1–51.2)	31.6 (25.9–38.1)	38.2 (21.6–58.2)	..	30.4 (13.8**–**44.7)	7.2 (3.7**–**10.2)
**Nyong et Mfoumou**	15.2 (12.0–19.0)	24.0 (11.6–41.9)	29.6 (25.4–34.1)	31.3 (22.2–41.2)	33.8 (28.0–40.7)	43.1 (24.9–63.4)	..	23.4 (6.6**–**38.7)	4.1 (1.4**–**6.6)
**Nyong et So'o**	32.6 (26.8–38.9)	38.8 (21.3–58.0)	26.3 (22.3–30.7)	18.7 (10.8–28.0)	55.3 (48.2–62.6)	67.2 (45.7–83.9)	21.0	11.3 (− 8.5**–**29.2)	6.9 (1.0**–**12.4)
**East**	**19.6 (15.6–24.2)**	**38.4 (21.5–57.3)**	**31.6 (26.5–37.3)**	**23.8 (14.4–37.4)**	**38.2 (30.9–46.0)**	**61.4 (39.8–79.1)**	**7.0**	**16.6 (**− **3.4–34.4)**	**35.7 (3.9–64.0)**
**Boumba et Ngoko**	17.3 (12.9–21.8)	66.1 (48.1–80.5)	40.3 (34.2–46.7)	12.6 (6.2–22.4)	30.1 (23.0–37.0)	83.9 (69.3–92.7)	90.0	4.6 (− 13.6**–**21.0)	2.6 (− 1.6**–**6.3)
**Haut Nyong**	15.8 (12.5–19.9)	36.1 (20.3–56.1)	27.0 (22.9–31.0)	18.9 (11.5– 27.8)	36.9 (30.5–43.9)	65.4 (44.8–82.0)	15.0	13.6 (− 5.9**–**31.0)	3.6 (− 1.2**–**8.0)
**Kadey**	17.3 (13.6–21.8)	42.8 (25.7–60.6)	29.7 (24.8–34.8)	23.2 (14.0–36.2)	36.9 (30.0–44.1)	64.5 (44.5–80.5)	11.0	16.0 (− 5.0**–**33.6)	9.0 (0.2**–**16.5)
**Lom et Djerem**	19.2 (14.8–24.1)	34.0 (19.0–53.0)	30.6 (25.6–37.0)	24.2 (15.5–37.8)	38.5 (30.3–46.4)	57.9 (37.2–76.0)	4.0	17.7 (− 2.3**–**35.2)	17.5 (3.8**–**29.5)
**Far North**	**5.1 (4.0–6.5)**	**10.8 (4.8–22.5)**	**23.1 (19.5–27.2)**	**23.2 (15.9–32.4)**	**18.0 (14.2–22.7)**	**31.8 (16.5–52.2)**	**..**	**18.2 (6.9–30.4)**	**164.3 (73.5–263.0)**
**Diamaré**	8.8 (6.9–11.2)	13.6 (6.2–26.6)	27.4 (23.1–32.0)	23.8 (16.7–32.9)	24.4 (19.4–30.1)	36.2 (19.7–55.8)	..	18.7 (6.1**–**31.8)	33.5 (13.6**–**54.5)
**Logone et Chari**	4.9 (3.8–6.3)	14.9 (6.5–29.5)	22.9 (19.2–27.1)	22.3 (15.1–31.5)	17.6 (13.5–22.5)	39.9 (21.0–60.9)	..	18.8 (6.1**–**32.5)	44.1 (20.4**–**70.7)
**Mayo Danay**	2.9 (2.2–3.8)	7.0 (3.0–14.8)	20.4 (16.9–24.3)	20.5 (13.9–29.4)	12.4 (9.2–16.4)	25.3 (12.2–44.5)	..	16.0 (7.5**–**25.9)	18.6 (9.2**–**29.6)
**Mayo Kani**	3.0 (2.3–3.9)	8.4 (3.7–18.2)	23.1 (19.3–27.4)	22.4 (15.3–31.0)	11.4 (8.6–15.0)	27.5 (13.4–47.4)	..	18.0 (8.1**–**28.8)	14.4 (6.9**–**22.7)
**Mayo Sava**	3.4 (2.6–4.3)	6.0 (2.6–13.3)	23.2 (19.5–27.7)	21.6 (14.5–30.5)	12.7 (9.6–16.4)	21.9 (10.2–40.6)	..	17.0 (8.8**–**26.7)	9.2 (4.8**–**14.5)
**Mayo Tsanaga**	4.3 (3.3–5.7)	15.9 (7.2–30.9)	18.2 (15.0–21.8)	22.9 (15.7–32.2)	19.1 (14.8–24.4)	40.8 (21.7–62.1)	..	19.4 (6.0**–**33.1)	48.0 (20.0**–**77.3)
**Littoral**	**21.1 (16.6–25.9)**	**26.4 (13.5–44.1)**	**35.2 (29.9–41.0)**	**28.7 (19.8–40.3)**	**37.5 (30.1–45.0)**	**47.5 (28.7–66.3)**	**..**	**22.7 (4.4–39.3)**	**292.4 (132.2–440.2)**
**Moungo**	15.3 (11.6–19.4)	27.2 (14.0–44.8)	40.7 (35.5–46.4)	33.3 (23.7–44.8)	27.3 (20.9–33.6)	44.5 (26.3–63.4)	..	26.0 (7.7**–**41.6)	11.2 (3.2**–**18.0)
**Nkam**	21.9 (17.4–26.9)	35.6 (19.4–54.8)	38.4 (33.4–43.6)	26.0 (16.9–35.7)	36.3 (29.8–43.4)	57.3 (37.5–75.6)	3.0	18.6 (− 0.3**–**35.7)	0.6 (− 0.1**–**1.2)
**Sanaga Maritime**	18.1 (14.3–22.6)	17.3 (8.0–32.5)	33.9 (29.5–38.8)	34.6 (24.9–45.5)	34.9 (28.4–41.4)	33.1 (17.3–53.3)	..	26.0 (10.4**–**40.3)	6.1 (2.7**–**9.4)
**Wouri**	22.0 (17.5–26.8)	26.2 (13.4–44.0)	32.8 (27.9–38.6)	28.1 (19.2–39.2)	40.2 (32.2–47.5)	48.0 (29.1–67.1)	..	21.7 (3.4**–**38.4)	268.5 (120.5**–**405.4)
**North**	**8.2 (6.5–10.3)**	**10.8 (4.9–22.6)**	**25.0 (21.0–29.1)**	**22.2 (15.6–30.8)**	**24.7 (19.9–30.1)**	**32.7 (17.0–52.9)**	**..**	**17.5 (6.7–29.9)**	**174.5 (89.5–276.1)**
**Bénoué**	8.1 (6.4–10.4)	14.3 (6.5–27.9)	26.8 (22.6–31.2)	24.2 (16.8–33.3)	23.4 (18.5–29.0)	36.9 (20.3–57.2)	..	19.1 (6.4**–**32.7)	132.2 (68.0**–**204.1)
**Faro**	4.4 (3.4–5.7)	4.9 (2.1–10.8)	26.4 (22.3–31.2)	20.9 (13.9–29.8)	14.3 (11.0–18.4)	18.9 (8.4–36.5)	..	16.2 (8.5**–**25.0)	1.0 (0.5**–**1.6)
**Mayo Louti**	3.9 (3.0–5.0)	8.0 (3.5–17.8)	21.3 (17.7–25.3)	19.0 (12.9–27.7)	15.4 (11.7–19.7)	29.3 (14.3–50.4)	..	15.4 (6.6**–**26.0)	27.9 (14.3**–**45.1)
**Mayo Rey**	3.1 (2.4–4.1)	10.3 (4.5–21.2)	25.1 (21.0–29.7)	21.0 (14.3–29.6)	11.1 (8.4–14.9)	32.9 (16.8–53.2)	..	16.6 (6.5**–**28.0)	22.5 (10.7**–**36.0)
**Northwest**	**25.2 (20.7–30.4)**	**30.1 (16.0–48.6)**	**29.7 (25.4–34.6)**	**25.4 (17.1–35.1)**	**45.8 (38.6–53.2)**	**53.9 (35.2–72.5)**	**1.0**	**18.5 (0.1–35.3)**	**63.4 (16.4–106.6)**
**Boyo**	0.1 (0.1–0.3)	0.7 (0.1–2.5)	36.2 (29.4–44.0)	40.1 (27.3–56.4)	0.4 (0.2–0.8)	1.6 (0.4–5.5)	..	30.4 (20.1**–**43.2)	4.6 (3.0**–**6.5)
**Bui**	21.5 (17.1 –26.5)	27.7 (14.4–46.5)	24.6 (20.9–29.0)	21.3 (14.0–29.9)	46.6 (39.1–54.0)	56.1 (36.1–75.1)	3.0	16.0 (− 1.2**–**32.5)	9.8 (2.0**–**17.5)
**Donga Mantung**	9.9 (7.7–12.4)	25.7 (12.6–44.5)	29.9 (25.4–34.6)	25.1 (17.0–34.4)	24.8 (19.8–30.7)	50.2 (30.4–70.6)	1.0	21.1 (4.2**–**37.1)	8.7 (2.5**–**14.5)
**Menchum**	0.1 (0.1–0.2)	0.4 (0.1–1.3)	28.7 (22.6–35.9)	28.6 (18.9–41.2)	0.4 (0.2–0.9)	1.2 (0.3–4.6)	..	21.6 (14.0**–**31.0)	5.3 (3.4**–**7.6)
**Mezam**	32.6 (26.5–38.9)	38.7 (22.6–57.0)	27.5 (22.3–34.0)	22.5 (14.3–33.6)	54.1 (45.4–62.4)	62.8 (42.5–78.9)	8.0	14.7 (− 4.7**–**32.2)	28.8 (9.3**–**46.2)
**Momo**	7.3 (4.5–11.4)	13.0 (4.6–30.5)	31.9 (25.5–38.9)	31.8 (22.1–43.3)	18.7 (12.0–27.3)	29.1 (12.3–52.3)	..	25.0 (5.1**–**43.4)	3.6 (0.6**–**6.4)
**Ngo Ketunjia**	30.3 (24.9–36.1)	32.7 (17.1–52.4)	31.4 (27.0–36.1)	29.8 (19.0–40.8)	49.1 (41.8–56.2)	52.0 (32.0–72.6)	1.0	21.6 (2.7**–**37.5)	4.2 (0.8**–**7.0)
**West**	**20.3 (16.1–24.9)**	**25.9 (13.0–43.5)**	**32.0 (26.8–37.8)**	**28.0 (19.1–39.7)**	**38.9 (31.6–46.4)**	**47.9 (28.9–66.4)**	**..**	**21.9 (3.5–38.5)**	**74.5 (21.0–123.3)**
**Bamboutos**	28.1 (22.6–34.6)	38.6 (22.1–57.0)	33.3 (27.8–39.7)	26.9 (17.0–38.7)	45.8 (37.3–54.2)	58.8 (38.3–76.2)	4.0	18.6 (− 0.6**–**35.0)	8.7 (0.7**–**15.6)
**Haut Nkam**	20.6 (15.6–26.2)	23.8 (11.8–40.5)	35.6 (29.1–43.4)	33.9 (23.5–46.5)	36.6 (27.6–45.5)	41.2 (23.4–60.8)	..	26.3 (8.2**–**42.6)	4.2 (1.2**–**6.8)
**Hauts Plateaux**	1.4 (1.0–2.1)	0.3 (0.1–0.7)	36.0 (31.0–41.4)	26.1 (18.1–36.0)	3.8 (2.6–5.5)	1.0 (0.3–2.9)	..	19.7 (13.5**–**27.2)	0.9 (0.6**–**1.3)
**Koung Khi**	18.0 (13.8–22.8)	21.2 (10.0–38.2)	36.2 (30.3–42.1)	30.6 (21.4–42.6)	33.2 (25.8–40.9)	40.7 (22.9–60.6)	..	24.5 (6.9**–**40.7)	0.6 (0.0**–**1.2)
**Menoua**	26.3 (20.8–32.2)	34.3 (18.8–52.3)	32.9 (27.1–39.9)	27.1 (17.9–38.9)	44.5 (35.5–53.1)	55.5 (35.8–73.4)	2.0	19.9 (0.9**–**36.4)	5.3 (− 0.3**–**10.2)
**Mifi**	23.6 (18.3–29.2)	35.1 (19.5–54.2)	35.6 (29.8–42.0)	25.6 (16.7–37.3)	39.8 (31.4–48.0)	57.4 (37.9–75.5)	3.0	19.9 (0.8**–**36.7)	24.8 (9.2**–**38.5)
**Ndé**	16.1 (12.1–20.7)	37.4 (20.7–56.8)	28.2 (23.1–34.5)	22.5 (14.0–32.5)	36.5 (27.5–44.7)	62.3 (41.6–79.6)	9.0	17.8 (− 1.0**–**34.4)	2.0 (0.0**–**3.8)
**Noun**	10.2 (7.5–13.5)	20.1 (9.7–36.2)	25.7 (21.6–30.6)	25.7 (17.7–36.5)	28.4 (21.7–35.6)	43.7 (25.5–62.6)	..	20.5 (3.8**–**37.0)	24.4 (7.3**–**41.4)
**South**	**21.6 (17.8–26.1)**	**20.3 (9.8–37.5)**	**31.2 (27.1–35.4)**	**29.7 (21.3–39.9)**	**40.9 (35.0–47.2)**	**40.2 (22.9–60.7)**	**..**	**22.6 (6.9–37.6)**	**54.3 (26.1–81.6)**
**Dja et Lobo**	13.7 (10.8–17.3)	11.9 (5.3–24.0)	30.2 (25.9–35.1)	29.6 (20.7–39.7)	31.2 (25.5–37.5)	28.7 (14.5–48.5)	..	22.8 (10.1**–**35.7)	8.1 (4.0**–**12.4)
**Mvila**	23.7 (19.3–29.0)	30.7 (15.8–50.3)	31.0 (26.8–35.7)	30.0 (19.3–41.2)	43.4 (36.6–50.5)	50.3 (30.1–71.2)	1.0	21.3 (2.8**–**37.0)	20.9 (9.8**–**30.4)
**Océan**	13.3 (10.2–17.0)	12.9 (5.7–26.3)	42.4 (37.0–47.9)	40.7 (30.7–52.1)	24.0 (18.7–29.9)	24.0 (12.0–41.8)	..	32.2 (17.8**–**45.1)	26.7 (16.9**–**35.8)
**Vallée du Ntem**	22.5 (18.3–27.7)	34.3 (18.3–53.7)	25.8 (22.0–30.1)	24.6 (15.6–34.7)	46.6 (39.8–53.8)	57.9 (37.7–76.5)	4.0	17.9 (− 1.1**–**34.5)	4.4 (0.9**–**7.4)
**Southwest**	**22.4 (17.9–27.4)**	**26.2 (13.4–44.3)**	**31.9 (27.4–37.3)**	**28.2 (19.5–38.9)**	**41.2 (33.8–48.4)**	**47.9 (29.8–67.4)**	**..**	**21.6 (3.2–37.8)**	**66.8 (21.4–106.8)**
**Fako**	20.1 (15.9–24.9)	22.8 (11.5–40.1)	30.6 (25.8–36.0)	29.8 (21.3–40.4)	39.5 (32.1–47.0)	43.1 (25.6–62.8)	..	22.4 (5.6**–**38.3)	36.0 (15.6**–**55.5)
**Koupé Manengouba**	29.0 (19.5–39.2)	51.0 (28.7–71.6)	35.3 (26.8–47.3)	18.7 (9.2–32.4)	45.0 (30.8–58.3)	72.9 (49.5–88.4)	42.0	12.6 (− 16.4**–**38.1)	0.5 (− 2.4**–**3.1)
**Lebialem**	17.3 (10.8–25.4)	35.9 (15.7–60.6)	30.0 (22.9–40.0)	17.1 (9.4–30.3)	36.5 (23.7–48.9)	67.1 (39.8–85.3)	23.0	15.0 (− 15.4**–**42.3)	2.7 (− 2.2**–**7.2)
**Manyu**	30.6 (21.6–40.6)	47.4 (25.0–70.3)	32.4 (25.7–40.7)	17.5 (8.5–31.0)	48.5 (36.3–60.0)	72.7 (47.3–88.8)	41.0	10.7 (− 20.5**–**37.5)	6.6 (− 3.9**–**15.5)
**Meme**	24.3 (19.4–29.7)	34.3 (18.4–53.5)	28.0 (23.8–33.2)	20.8 (13.1–30.1)	46.4 (38.6–53.7)	62.0 (41.9–79.3)	9.0	16.0 (− 2.9**–**32.8)	11.5 (1.3**–**20.6)
**Ndian**	21.0 (13.5–30.0)	34.3 (15.5–57.2)	35.3 (26.8–45.7)	28.7 (16.5–46.7)	37.2 (24.6–50.5)	53.9 (28.0–76.5)	4.0	22.2 (− 8.1**–**46.2)	1.9 (− 1.3**–**4.5)

Estimates are in % (95% credible interval); modern methods refer to modern contraceptive methods; .. = 0% attainment probability. Slight discrepancies between higher vs combined lower administrative unit estimates stem from differences in observations (data points) and separate runs of the FPET model per unit. Even though, statistical tests (*p* < 0.05) showed no significant differences (Additional File 3: Table S5)

The 2015 divisional-level estimates of mCPR and demand satisfied with modern methods, respectively, ranged from as high as 32.6% (26.8 to 38.9) and 55.3% (48.2 to 62.6) in *Nyong et So'o* division, *Center* region, to as low as 0.1% (0.1 to 0.3) and 0.4% (0.2 to 0.8) in *Boyo* division, *Northwest* region. In the same year, unmet need for modern methods ranged from the lowest at 18.2% (15.0 to 21.8) in the *Mayo Tsanaga* division, *Far North* region, to the highest at 48.4% (43.6 to 53.4) in the *Nyong et Kéllé* division, *Center* region (Table 1). 2030 divisional-level projections for mCPR and demand satisfied with modern methods show distinguishingly higher levels of 66.1% (48.1 to 80.5) and 83.9% (69.3 to 92.7) in *Boumba et Ngoko* division, *East* region, where the lowest levels of unmet need for modern methods, 12.6% (6.2 to 22.4), are also expected. Additional File 3 (Table S6) includes estimated numbers of married women of reproductive age using, with unmet need, and demand satisfied for modern contraception at the national, regional, and divisional levels.

**Fig. 2:**
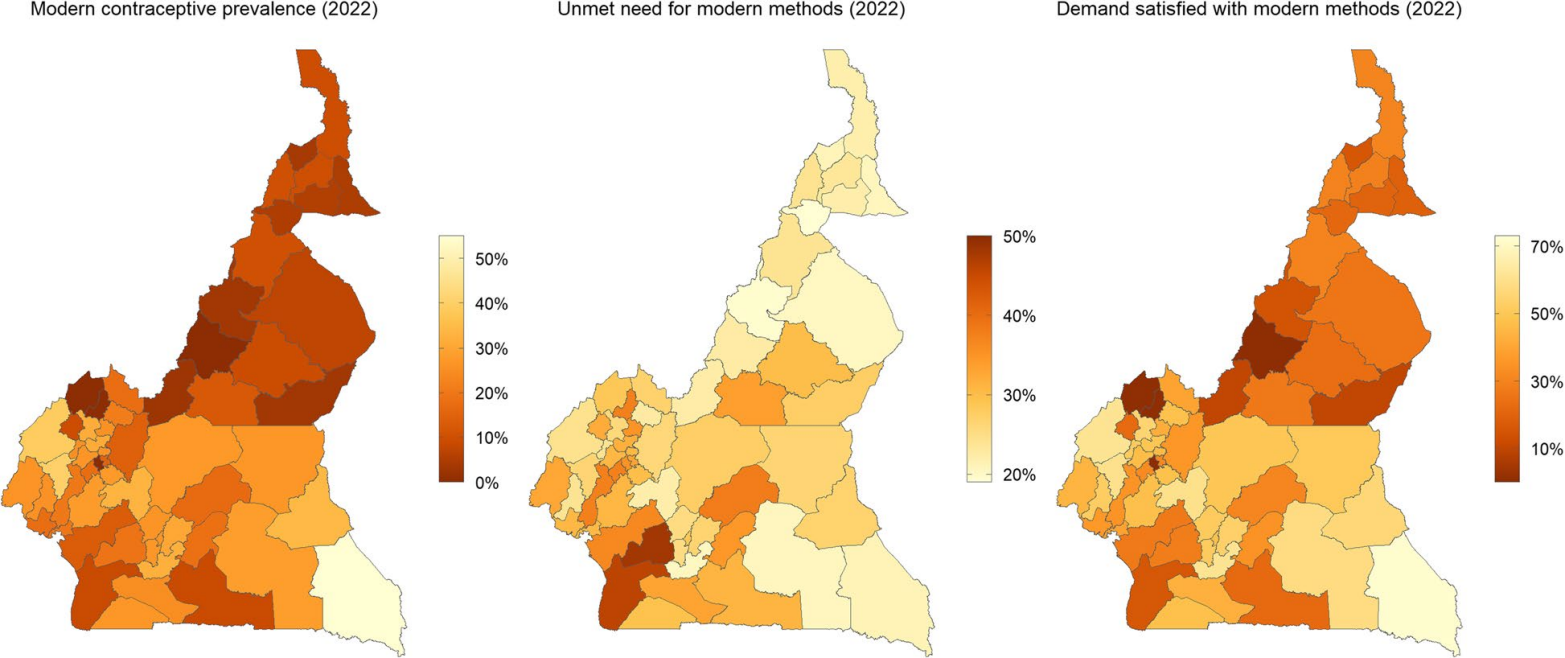
Estimates of use, unmet need, and demand satisfied for modern methods across Cameroon’s divisions (2022) Modern methods = modern contraceptive methods

Median estimates of all three indicators are presented geospatially in Fig. 2 for the year 2022. These show that northern divisions (i.e., divisions within the *Adamawa*, *Far North*, and *North* regions) had the lowest levels of favourable indicators, with the majority recording mCPR of ≤ 10.3% and demand satisfied with modern methods of ≤ 30.1%. Conversely, most of these divisions also recorded the lowest levels of the adverse indicator, i.e., unmet need for modern methods, with *Mayo Louti* division having the lowest at 19.1% (15.1 to 23.9), followed by *Faro* with 19.4% (15.2 to 24.3), and *Mayo Rey* with 20.4% (16.1 to 25.6). These three divisions are located within the *North* region. Meanwhile, rates of unmet need for modern methods were highest in the southern divisions of *Nyong et Kéllé*, 48.1% (41.8 to 54.1) and *Océan*, 46.0% (39.4 to 53.1).

### Trends and projections of family planning

**Fig. 3:**
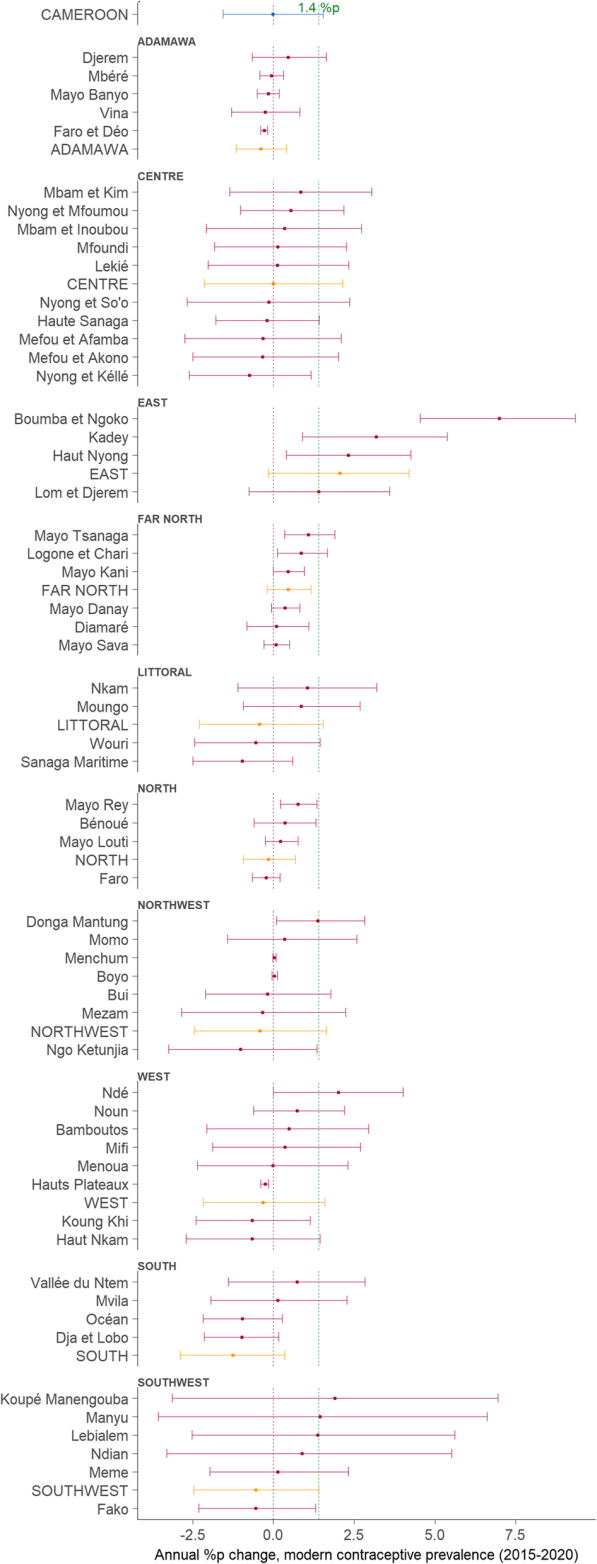
Annual change in modern contraceptive prevalence for the regions and divisions of Cameroon, 2015–2020 %p = percentage points; points = median; horizontal lines = 95% credible intervals; Plots, blue = country-level, orange = regions, red = divisions; red and green dotted line = mark for 0·0 and 1 4 %p change, respectively

Figure 3 illustrates the trends in mCPR in terms of annual changes between 2015 and 2020. During this period, Cameroon registered a yearly trend of − 0.02%p (− 1.6 to 1.54). However, only the *Boumba et Ngoko* division in the *East* region attained the proposed annual increase in mCPR of ≥ 1.4%p, lower and upper CrIs inclusive [7.0%p (4.5 to 9.3)]. Overall, only around one-sixth of the divisions, including all four divisions within the *East* region, attained the ≥ 1.4%p target according to median estimates. It is worth noting that up to around one-third of the divisions displayed an annual change in mCPR of ≤ 0%p, indicating a decrease in levels. The *East* region alone, with an estimated annual change in mCPR of 2.1%p (− 0.2 to 4.2), recorded a median growth rate meeting or exceeding the 1.4%p target. In contrast, all other regions, except for the *Far North* with an annual rate of 0.5%p (− 0.2 to 1.2), exhibited declining mCPR trends based on the median estimates.

Table 1 also presents the attainment probabilities of and increases in users of modern contraceptives required to meet the proposed target of ≥ 75% demand satisfied in 2030. Only around one-third of the regions and less than half of the divisions have some probability of attaining this target. *Boumba et Ngoko* division within the *East* region recorded the highest probability at 90.0%. This was distantly followed by *Koupé Manengouba* and *Manyu* divisions in the *Southwest* region, with probabilities of 42.0% and 41.0%, respectively. Based on figures from 2022, it is estimated that an increase of 21.4% (4.0 to 37.4) or 1.4 (0.6 to 2.2) million additional users of modern methods of contraception are required to meet the target of at least 75% demand satisfied with modern methods by 2030.

### Indirect effects of COVID-19/armed conflict on family planning projections

**Fig. 4:**
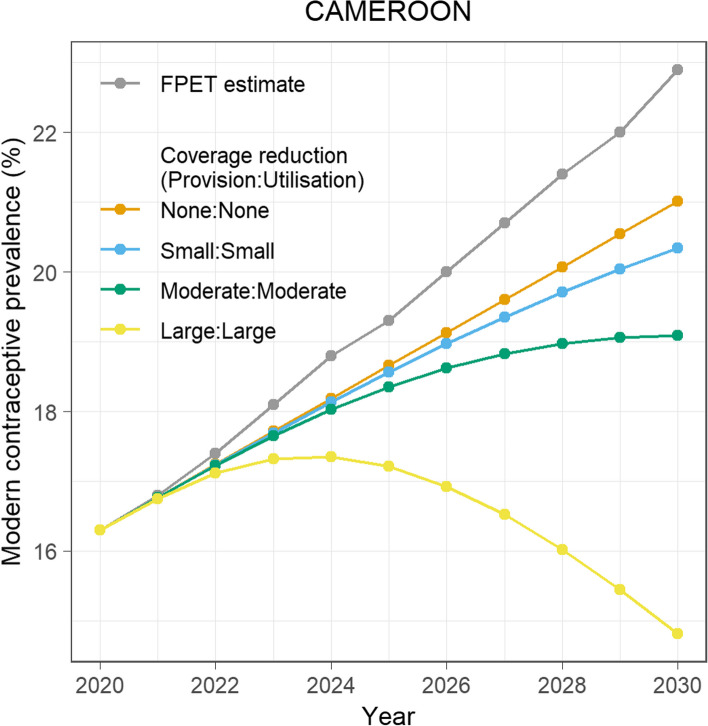
Modern contraceptive use given varied scenarios of services reductions in Cameroon, 2020–2030 FPET = Family planning estimation tool; None = 0%, Small = − 5%, Moderate = − 10%, and Large = − 25% annual reduction in coverage of services (i.e., either “Provision” or “Utilization”)

**Fig. 5:**
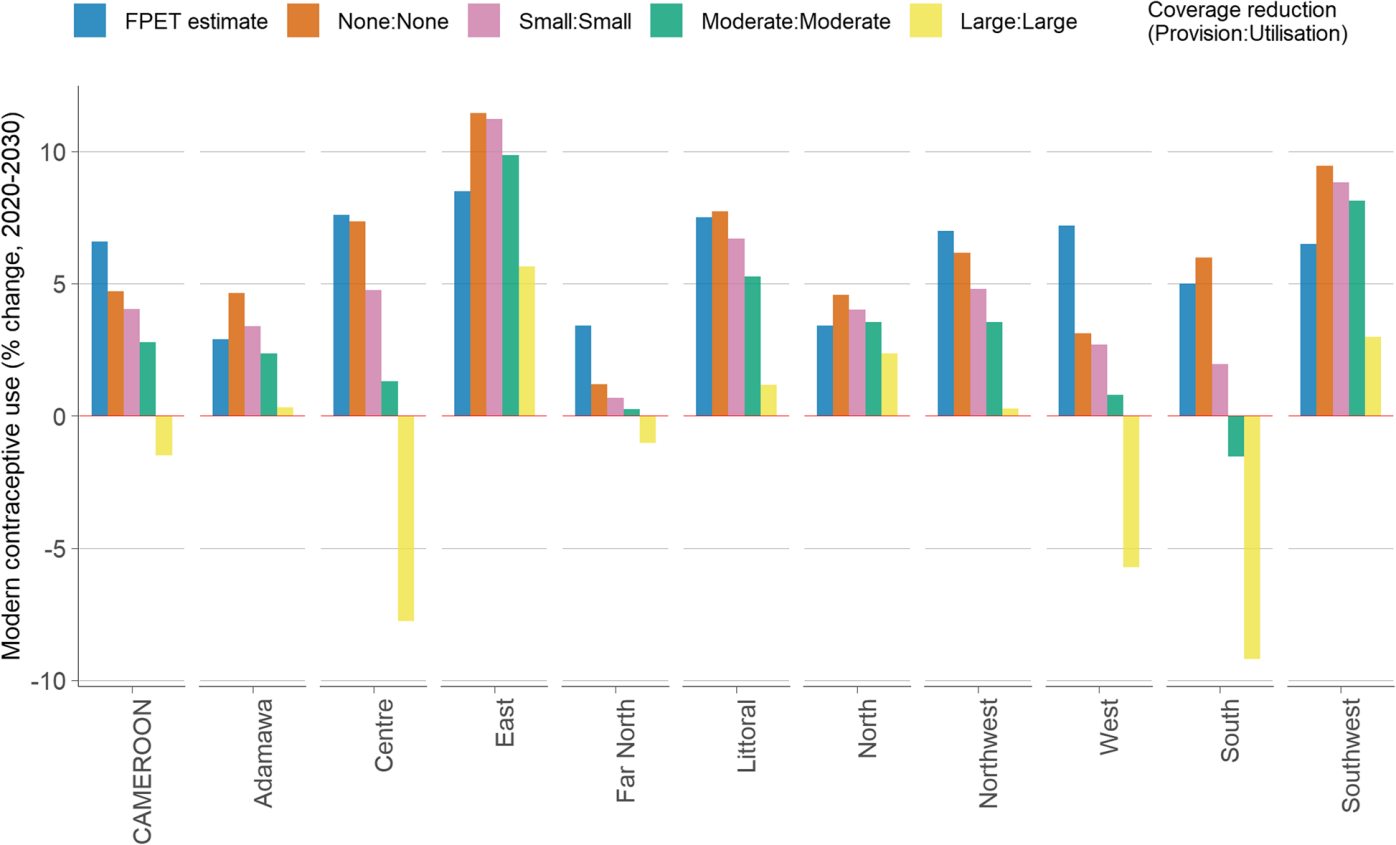
Modern contraceptive use given varied scenarios of services reductions across regions of Cameroon, 2020–2030 FPET = family planning estimation tool; None = 0%, Small = − 5%, Moderate = − 10%, and Large = − 25% annual reduction in coverage of services (i.e., either “Provision” or “Utilization”); Red line (0 on the y-axis) shows the cutoff point for levels of modern contraceptive prevalence in 2020

Lastly, results of possible effects on projections of mCPR given varied scenarios of reductions in services coverage due to unprecedented adverse events, such as COVID-19 and armed conflict, in Cameroon from 2020 to 2030 are presented. Figure 4 shows that from “None” (0%) to large (− 25%) annual changes in services coverage (i.e., either “Provision” or “Utilization”), steady contractions could be recorded in the overall projected levels of mCPR. Nationally, if there are “None” or “Small” (− 5%) annual changes in services coverage, the mCPR is expected to continue rising to an estimated 21.0% or 20.3%, respectively, by 2030. However, these figures are lower than the FPET projection of 22.9%. If there are “Moderate” (− 10%) annual reductions in services coverage, the mCPR could begin to level off from 2028 onwards. On the other hand, “Large” reductions in services coverage could result in a decline in mCPR from 2024 onwards, dropping below baseline levels of 16.3% in 2020 to 14.8% by 2030. Figure 5 displays the cumulative change in mCPR from 2020 to 2030 at the national and regional levels, considering various scenarios of reductions in services coverage. Across all regions, steady contractions in the projected levels of mCPR were observed given reductions in service coverage ranging from “Small” to “Large.” Except for the *East* and *Southwest* regions, only when reductions in services coverage reach “Moderate” levels (i.e., − 10% per annum) do the cumulative mCPR projections drop below those from the FPET. In the *South* region, negative trends or an mCPR 1.5% below baseline levels could be recorded given “Moderate” reductions in services coverage. Nationally, as well as in the *South*, *Center*, and *West* regions, large reductions in services could reverse the projected trajectories for mCPR, causing contractions of up to − 1.5%, − 9.2%, − 7.8%, and − 5.7%, respectively, below the figures projected by the FPET. Additional File 3 (Table S7) includes mCPR projections for all sixteen scenarios of services reductions per region.

## Discussion

Overall, in Cameroon and across its regions and most of its divisions, continuous improvements were observed in favourable indicators, i.e., mCPR and demand satisfied with modern methods. Additionally, there were steady decreases in the adverse indicator, i.e., unmet need for modern methods. The improvements in demand satisfied with modern methods were generally much larger than in mCPR. This indicates that the increase in modern contraceptive use is faster among those with a demand for family planning compared to the overall population of married women of reproductive age. These findings are not unexpected, as knowledge and use of contraceptive methods were reported to have risen considerably among both Cameroonian men and women of reproductive age [49].

Conspicuous subnational variations were seen for all three family planning indicators. The widest disparities were seen in terms of demand satisfied with modern methods. These disparities were particularly notable between the *East* and *Adamawa* at the regional level, and between *Boumba et Ngoko* and *Hauts-Plateaux* at the divisional level. The *East* region and its *Boumba et Ngoko* division stand out in terms of remarkable improvements as they have recorded significant increases in demand satisfied with modern methods and decreases in unmet need for modern methods. These successes could be attributed to several factors. In recent decades, this region became a focal point for the roll-out of common barrier contraceptives like male/female condoms following a sharp rise in HIV infection rates, which were the highest in the country [49]. The findings may also be related to the size of the differences in data inputs between earlier and later surveys. For instance, when comparing the earliest and latest survey data, the *East* region showed the highest absolute difference in mCPR (+ 22.6%), with mCPR increasing from 5% in the 1991 DHS to 27.6% in the 2018 DHS [50]. In contrast, lower-performing regions like the *Adamawa*, *Far North*, and *North* recorded only modest increases of + 5.1, + 5.8, and + 6.0%, respectively, during the same period. Nonetheless, the most recent survey reflected sharp improvements in the *East* region for all three family planning indicators [49].

Remarkably, for the three northern regions (*Adamawa*, *Far North*, and *North*) and their divisions, the unmet need for modern methods has remained low, even though their mCPRs are among the lowest. Low unmet need could be observed in cases where mCPR is very low, indicating a corresponding low total demand. Notably, previous assessments revealed relatively low demand for family planning in the said regions [49]. However, a country-specific assessment of systematic trends demonstrated that when there is a trajectory of increasing contraceptive prevalence, unmet need tends to initially increase [51]. A decrease in unmet need sets in when the contraceptive prevalence rate increases to between 20 and 60%, indicating that use is increasing faster than demand. Trajectories in the above regions are unclear considering that results in terms of annual changes in mCPR between 2015 and 2020 were variable, ranging from − 1.16 to 1.16%p. Regardless, the sustained low levels of unmet need for modern methods despite low mCPRs suggest that locality-specific factors could be of influence. The low levels of unmet need could be related to low demand for family planning, which can be attributed to dominant sociocultural factors inherent in the northern regions, such as religion (mainly Islam) and tribal customs (mainly Hausa), that inherently encourage marriage, including polygamy and childbearing [52]. Additionally, a higher rate of illiteracy, reaching up to 76.0% in some parts [53], could be a contributor.

Divisional-level estimates of mCPR reveal particularly low figures in the *Boyo* and *Menchum* divisions in the *Northwest* region, and the *Hauts Plateaux* division in the *West* region. Additionally, unmet need for modern methods is highest in the *Nyong et Kéllé* division, *Center* region, and *Océan* division, *South* region. The reasons behind these discrepancies remain unclear, but the ongoing armed conflict in the English-speaking *Northwest* and *Southwest* regions [19], where *Boyo* and *Menchum* divisions are located, likely plays a role. The civil conflict, which began in 2016, led to the destruction of health facilities and the displacement of healthcare personnel, severely impacting routine service delivery, including family planning. Furthermore, heightened fighting in certain areas may have affected data collection during the most recent survey (DHS-2018) [49], in which case actual post-conflict trends may not be fully reflected. Nonetheless, the long span of the exploited data (1991 to 2018) and data from pre-conflict surveys (1991 to 2011) consistently showing low mCPR in these same locations, suggests that the estimated figures may not be highly disparate.

Regarding defined targets, wide variabilities were also seen. This study’s estimate of 0.6 (0.4 to 0.8) million modern contraceptive users in 2020 falls markedly below the 3.8 million that would have been if the recommended 1.4%p annual increases toward FP2020 [1] had been achieved. Still, moving forward, figures could greatly vary especially given disparities in subnational trends. By regions, for instance, because the credible intervals recorded for annual changes in modern contraceptives are quite wide, the attainability of at least a positive yearly increase (≥ 0%p) or even the 1.4%p target in modern contraceptive use cannot be clearly stated. The same is the case for demand satisfied with modern methods, where per the estimated margins across the regions, the probability of attaining the target of ≥ 75% in 2030 remains unclear. As New et al*.* (2017) [8] suggest, defining trend-based attainment probabilities instead would allow for setting more realistic targets per subnational unit. Even so, to reduce the subnational gaps, the government should direct more resources to areas that are lagging, especially in terms of the unmet need for modern methods. Strategies should likewise stimulate demand for long-acting reversible contraceptives (LARCs) such as intrauterine devices and subdermal implants that are more effective birth controls [54, 55]. Such a strategy will suit Cameroon as a recent study reported a low prevalence (8.0%) in the utilization of LARCs [55]. Sharp increases in mCPR in several countries within sub-Saharan Africa have been attributed to rises in the use of LARCs. Cameroon already initiated community-based projects for the distribution of three newly introduced LARCs in 2015 [6]. Notwithstanding, it would be crucial to train more qualified service providers to add to the limited numbers [56], especially in areas where secure and reliable contraceptive delivery/supply mechanisms are lacking.

Furthermore, considering disruptions to healthcare delivery due to the COVID-19 pandemic amidst the ongoing conflict in Cameroon, we examined the likely effects on projections of family planning. Our findings indicate that projected increases in family planning at the national and regional levels could be significantly reduced depending on the extent to which services coverage is constrained by these events. Varying percentages of contractions in projections of modern contraceptive use were observed across the different regions, also highlighting regional inequalities in the provision of services per capita. For example, in the *Centre*, *West*, and *South* regions where the largest likelihoods of impairments to modern contractive use were recorded, health services coverage per capita is relatively higher compared to the other regions [41]. However, the impact of services coverage disruptions on projections of modern contraceptive use may actually be highest in the harder-hit conflict zones such as the *Northwest* and *Southwest* regions, as well as nearby areas like the *West* and *Littoral* [19]. It is important to note that the provision of family planning services relies on a robust network of supplies, communication, and qualified personnel. Any disruptions to this chain, whether due to disease outbreaks or conflicts, can greatly impair both the capacity and quality of service delivery. Therefore, family planning strategies must be adaptive and resilient to emergencies or disruptions to ensure continuity of services. It is worth mentioning that if our analysis had included log-transformed raw mCPR projections (i.e., from the FPET), even the largest reductions in services coverage would, at most, result in the current levels of family planning being maintained. However, current levels wouldn't be guaranteed due to the unpredictable nature of the impact of such events [15, 17]. Additionally, it is important to acknowledge that the assumptions made for the impact assessment are only tentative and could significantly vary based on other factors that were not incorporated into the analysis.

A primary contribution of this study is the characterisation of family planning indicators at a subnational and demographic subgroup scale, which can facilitate policy-making by health officials. Evaluating progress among married women of reproductive age, a subgroup that is of key interest in family planning services provision, can inform decisions on the allocation and long-term scaling of family planning resources accordingly with the country’s administratively aligned healthcare system [14]. Furthermore, including counts of those with and without access to modern methods (Additional File 3: Table S6) would quantitatively inform the targeting or prioritization of those in the least performing regions and divisions. For instance, areas with a low prevalence of modern contraceptive use but higher numbers with unmet need could be prioritized over those with a higher prevalence of use but lower numbers with unmet need. This study also underscores the significance of robust survey data in analyses of health performance for smaller areas. Also, the methods and analysis of the indirect effects of reductions in health services coverage can be adopted and/or generalized to other countries with similar data limitations. Other advantages stem from the characteristics of the FPEM, which, based on its hierarchical set-up makes it suitable as a standard tool for subnational assessments across different settings. Furthermore, employing the FPET allows for direct multi-country comparability within a global health context of universally defined targets (such as the FP2030 and SDGs), even at a subnational scale.

Notwithstanding the realizations of this study, it is not without limitations. Mainly, this relates to the unavailability and uncertainty associated with data for smaller areas (second-level administrative units). The limited data points present a challenge for measuring trends, especially for unmet need [45]. However, with the FPEM, these limitations are minimized by capturing changes in recently observed data and incorporating survey sample-specific uncertainties. Additionally, uncertainties surrounding estimates are wider and substantially increase for years further from the most recent observed data point. Secondly, no subnational predictor, such as service provision or the Estimated Modern Use, was incorporated due to data unavailability. Nonetheless, the time-dependent distortions capture population-specific changes in each indicator [57] and the estimates become more reliant on past trends [12]. Limitations of the FPEM set-up include the assumption that accelerated increases in contraceptive prevalence rate would stimulate similar changes in demand and unmet need. However, this should not have a major impact on the findings as unmet need was shown to follow similar trajectories of change with the contraceptive prevalence rate [45]. Lastly, this study only includes women who are married or in a union. While this is a standard reference group for evaluations [45], future analyses should include unmarried women as they constitute an increasing share of users of family planning services [58]. Furthermore, given the discrepancies in definitions of the demand for family planning between the two groups [44, 58], we aim to conduct a separate assessment for the unmarried population.

## Conclusions

This study uncovers subnational variabilities in core family planning indicators across Cameroon. Estimates of modern contraceptive use were 79.0 to 89.5% less than the FP2020 target for 2020, and projections for 2030 indicate that the proposed SDG of ≥ 75% demand satisfied with modern methods could be attained in none to as few as one-third of the 58 divisions. Unprecedented events disrupting health services coverage, such as the COVID-19 pandemic and armed conflict in Cameroon, could limit projected increases in modern contraceptive use across all 10 regions of the country. The identified gaps should facilitate the targeting of particularly lagging populations, such as in the *Boyo* division, while also replicating successes seen in divisions like *Boumba et Ngoko* as efforts toward SDG 3.7 continue.

## Supplementary Information

** Additional file 1.** Data and Variables. ▪ Data (*Family planning data*: ⇒ Table S1: Characteristics of data used to analyse family planning indicators in Cameroon; *Population data*: ⇒ Estimation of populations of married women of reproductive age. ⇒ Estimation of populations of unmarried women of reproductive age. ⇒ Table S2: Variables and data used to estimate married women of reproductive age counts sub-nationally in Cameroon). ▪ Variables (⇒ Table S3: Definitions of family planning indicators).

**Additional file 2.** Modelling Indirect Effects of Services Disruptions on Family Planning Projections. ⇒ Figure S1: Framework for the effects of health system components on coverage of health services. ⇒ Formula translating component reductions to overall change in family planning outcomes. ⇒ Table S4: Regression of components of services coverage against family planning indicators.

**Additional file 3.** Supplementary Results. ⇒ Table S5: *t*-test results comparing the aggregates of subnational- to the national-level FPET estimates for Cameroon from 1990–2030. ⇒ Table S6: Counts of married women using, with unmet need, and demand satisfied for modern contraception across Cameroon’s regions and divisions in 2015 and projections for 2030. ⇒ Table S7: Projected levels of modern contraceptive use given varied percentage annual reductions in services coverage by regions of Cameroon, 2020–2030.

## Data Availability

The datasets supporting the conclusions of the FPET analysis are publicly available from Demographic and Health Surveys (https://dhsprogram.com/methodology/survey-search.cfm?pgtype=main&SrvyTp=country&ctry_id=4) conducted in 1991 [23], 1998 [24], 2004 [25], 2011 [26], and 2018 [27], and Multiple Indicator Cluster Surveys (https://mics.unicef.org/surveys) conducted in 2000 [28], 2006 [29], and 2014 [30]. Data and Codes for the COVID/Conflict-related analysis are now available online (https://github.com/RS-Nsashiyi/FamilyPlanning-COVID19_Conflict) [59].

## Abbreviations

FP2020: Family Planning 2020
FP2030: Family Planning 2030
COVID-19: Coronavirus disease 2019
SDG: Sustainable development goals
FPET: Family Planning Estimation Tool
FPEM: Family Planning Estimation Model
DHS: Demographic and Health Survey
MICS: Multiple Indicator Cluster Survey
QGIS: Quantum Geographic Information System
DMPA-SC: Subcutaneous depot medroxyprogesterone acetate
LARC: Long-acting reversible contraceptive
SMS: Short message service
mCPR: Modern contraceptive prevalence rate
HRH: Human resources for health
FH: Facilities for health
HB: Health budget
CrI: Credible interval
