# Supplementary material for: Contraceptive use, unmet need, and demand satisfied for family planning across Cameroon: a subnational study including indirect effects of COVID-19 and armed conflict on projections
Source: BMC Glob Public Health. 2024 Jul 3;2:40. doi: 10.1186/s44263-024-00071-4 (PMC11622886; doi:10.1186/s44263-024-00071-4)
Supplement: Supplementary file 1 — Additional file 1. Data and Variables. ▪ Data (Family planning data: ⇒ Table S1: Characteristics of data used to analyse family planning indicators in Cameroon; Population data: ⇒ Estimation of populations of married women of reproductive age. ⇒ Estimation of populations of unmarried women of reproductive age. ⇒ Table S2: Variables and data used to estimate married women of reproductive age counts sub-nationally in Cameroon). ▪ Variables (⇒ Table S3: Definitions of family planning indicators). [file 44263_2024_71_MOESM1_ESM.docx]

**Additional file 1**

**Data and Variables**

**Data**

**Family planning data**

**Table S1: Characteristics of data used to analyse family planning indicators in Cameroon**

| **Data source** | **Year** | **Sample size** | **Number of data points** | | | | |
| --- | --- | --- | --- | --- | --- | --- | --- |
|  |  |  | **Country** | | | | |
|  |  |  | ✓/✗ | Observations | | | |
|  |  |  |  | Contraceptive prevalence | | | Unmet need |
|  |  |  |  | modern | tradi | total |  |
| CFS | 1978 | ... | ✓ | 1 | 1 | 1 | 0 |
| DHS | 1991 | 3871 | ✓ | 1 | 1 | 1 | 1 |
| DHS | 1998 | 5501 | ✓ | 1 | 1 | 1 | 1 |
| MICS | 2000 | 5069 | ✓ | 1 | 1 | 1 | 0 |
| DHS | 2004 | 10656 | ✓ | 1 | 1 | 1 | 1 |
| MICS | 2006 | 9408 | ✓ | 1 | 1 | 1 | 1 |
| DHS | 2011 | 15426 | ✓ | 1 | 1 | 1 | 1 |
| MICS | 2014 | 10447 | ✓ | 1 | 1 | 1 | 1 |
| DHS | 2018 | 13500 | ✓ | 1 | 1 | 1 | 1 |
|  |  |  | **Regions** | | | | |
| DHS | 1991 | 3871 | ✗ | 0 | 0 | 0 | 0 |
| DHS | 1998 | 5501 | ✓ | 10 | 10 | 10 | 10 |
| MICS | 2000 | 5069 | ✓ | 10 | 10 | 10 | 10 |
| DHS | 2004 | 10656 | ✓ | 10 | 10 | 10 | 0 |
| MICS | 2006 | 9408 | ✓ | 10 | 10 | 10 | 10 |
| DHS | 2011 | 15426 | ✓ | 10 | 10 | 10 | 0 |
| MICS | 2014 | 10447 | ✓ | 10 | 10 | 10 | 10 |
| DHS | 2018 | 13500 | ✓ | 10 | 10 | 10 | 10 |
| DHS | 1991 | 3871 | ✓ | 10 | 10 | 10 | 10 |
|  |  |  | **Divisions** | | | | |
| DHS | 1991 | 3871 | ✗ | 0 | 0 | 0 | 0 |
| DHS | 1998 | 5501 | ✓ | 52 | 52 | 52 | 52 |
| MICS | 2000 | 5069 | ✗ (except *M*&*W*) | 2 | 2 | 2 | 2 |
| DHS | 2004 | 10656 | ✗ (except *M*&*W*) | 2 | 2 | 2 | 0 |
| MICS | 2006 | 9408 | ✓ | 58 | 58 | 58 | 58 |
| DHS | 2011 | 15426 | ✗ (except *M*&*W*) | 2 | 2 | 2 | 0 |
| MICS | 2014 | 10447 | ✓ | 58 | 58 | 58 | 58 |
| DHS | 2018 | 13500 | ✗ (except *M*&*W*) | 2 | 2 | 2 | 2 |
| DHS | 1991 | 3871 | ✓ | 51 | 51 | 51 | 51 |

CFS=Cameroon Fertility Survey; DHS=Demographic and Health Surveys; MICS=Multiple Indicator Cluster Surveys; ...=unspecified sample size; ✓=Yes; ✗=No; *M*=Mfoundi and *W*=Wouri divisions; tradi=traditional

For regional-level analysis, there were 80 observations each for modern, traditional, and total contraceptive prevalence rates, and 60 observations for unmet need for family planning. There were, respectively, 227 and 223 observations for divisional-level indicators. These were sourced from all but the Multiple Indicator Cluster Surveys (MICS) and Demographic and Health Surveys (DHS) 1998 via reclassification of data for each cluster based on geolocation, using the geographic information system (GIS) software QGIS 3.8 [1]. The *Mfoundi* (Yaoundé) and *Wouri* (Douala) divisions each had 18 more observations that were sourced from MICS and DHS 1998 as they were surveyed as separate regions. There were at least three data points per parameter for each administrative unit.

DHS and MICS are cross-sectional surveys on a nationwide scale with similar methodologies [2]. In Cameroon, a two-stage stratified random sampling technique to collect information on individuals within households. In the first stage, the enumeration areas (EA) from the general census of the population are sampled proportionally to the number of households in clusters after stratification in rural and urban EA respectively. The data are representative at national and regional levels. For the most recent DHS and MICs surveys, 580 sample points (or clusters) were randomly selected in the first stage. In the second stage, 24 households in each urban cluster and 28 households in each rural area were selected from each sample point in all regions. Approximately equal sample sizes are derived for each region. Therefore, weighting factors are added to the data file so that the estimates are self-weighting/proportional at the national level. Our data is based on the woman’s questionnaire that is used to collect information on all females aged 15 to 49 years.

## **Population data**

**Estimation of populations of married women of reproductive age**

Statistical analysis to estimate the population of married or in union women of reproductive age (MWRA) by single age 15–49 years, for each division and region of Cameroon, builds on standard regression forecasting approaches that assume factors influencing population size to have measured effects on population change over time [3, 4]. In this multiple regression approach, we expand upon these with reference to proposed Bayesian methodologies on small-area population forecasting [5, 6] incorporating as covariates, age-specific vital rates for fertility and survival, and net-migration proportion, which are demographic drivers that are considered to be of key interest to the dynamics of populations of females [7]. Age-specific estimates derived from fertility and net-migration models as well as survival probabilities interpolation were used (Table S2).

The model for population estimation was constructed [6, 8] such that for each age of MWRA, changes in the log-transformed population size of MWRA will be influenced by patterns of age-specific fertility and survival rates, and net-migration proportion for the corresponding ages within divisions and regions, and built in a time series approach to captures trends over time. The expected number of women aged *a* in division *d* within region *r* at time *t* was generated based on the general relation modelled on the log-scale as;

$$\log\eta_{adrt}^{*}=\beta_{0,adrt}+\beta_{1,adrt}.X_{1,adrt}+\beta_{2,adrt}.X_{2,adrt}.X_{3,adrt}+u_{1,adt} +u_{2,drt}+\varepsilon_{adrt}$$

Where; $\eta_{adrt}^{*}$ equals the expected number of MWRA age *a* in division *d* region *r* at time *t*; $\beta_{0,adrt}$ is the random intercept term; $\beta_{1,adrt}$ and $X_{1,adrt}$ are the coefficient and observation associated with the net-migration proportion for MWRA aged *a* in region *r* at time *t*; $\beta_{2,a,d,r,t}$ shows the coefficient for the interaction term fertility and survival, $X_{2,adrt}$ and $X_{3,adrt}$ are the observations associated with fertility and survival for WRA aged *a* in division *d* region *r* at time *t* ; $u_{1,adt}$is the random slope defined by age *a* and division *d* at time *t*, and $u_{2,drt}$ is the random slope set per division d and region *r* at time *t*; and $\varepsilon_{adrt}$ is the residual of the hierarchical model.

Standard regression forecasting model proceeds in two steps. In step one, the relationship between the dependent and independent variables is established by estimating the $\beta$ vectors [9]. This model established a set of relationships between changes in the population (dependent variable) with changes in the demographic drivers (independent variables) for each corresponding age, division, and region over time. In step one, time $(t)$ corresponds with the census years $t$, $t+11$, and $t+29$ representing observations for 1976, 1987, and 2005, respectively. In step two, out-of-sample population predictions for years from 1990–2030 that represent the periods between $t+14$ to $t+54$ were derived based on the relationships established in step one. These predictions incorporate values at $t+14$ to $t+54$ for each of the demographic drivers as estimated from their respective models. Importantly, the independent variables have to be approximately linearly related to the dependent variable and also not highly correlated [9]. Checks for linearity and intercorrelation gave *p* < 0.001 and *r* ≤ 0.62, respectively.

**Computations:** Best estimates for all parameters of interest for each model were computed as the median of generated samples from the posterior distributions via Markov Chain Monte Carlo (MCMC) algorithm. Final estimates of MWRA counts for each administrative unit and year are the corresponding aggregates of the median of posterior samples for the ages 15–49 years. 95% Credible Intervals equal the 2.5^th^ and 97.5^th^ percentiles of the samples. For each model, standard diagnostics were conducted to evaluate the convergence of MCMC, including visual observations of trace plots for when the outputs of chains overlapped [10], and quantitatively, the Gelman-Rubin diagnostics for values close to 1 and at most <1.1 [10]. This statistical analysis was conducted using Stata Statistical Software: Release 17.1.

**Estimation of populations of unmarried women of reproductive age**

The same model setup as outlined above for married women of reproductive age was used to generate population counts of unmarried women of reproductive age. However, the IPUMS International Census data subset of raw population counts and net-migration for the unmarried population was incorporated in this case. Fertility rates from DHS data and Survival probabilities from the WPP-life tables were maintained.

**Table S2. Variables and data used to estimate married women of reproductive age counts sub-nationally in Cameroon**

| Variables | Data | Year | Sample size | Statistical model | Equation |
| --- | --- | --- | --- | --- | --- |
| Net-migration count | IPUMS-International Census data | 1976 | 146,973 | BHM | $m_{adrt}^{*}=\beta_{0,adrt}+I_{1,adt} +I_{2,drt} + \varepsilon_{adrt}$…....... (A) |
|  |  | 1987 | 163,784 |  |  |
|  |  | 2005 | 295,785 |  |  |
| Age-specific fertility rate | DHS | 1991 | 3,871 | BHM | $\log f_{art}^{*}=\beta_{0,art}+z_{1,art}+ \varepsilon_{art}$………………… (B) |
|  |  | 1998 | 5,501 |  |  |
|  |  | 2004 | 10,656 |  |  |
|  |  | 2011 | 15,426 |  |  |
|  |  | 2018 | 13,527 |  |  |
| Age-specific survival probability | WPP-life tables | 1976–2030 | NA | Linear interpolation | Five-year probabilities of surviving between the ages *x* and *x + 5*, *5px*, were converted to single-age and single-year probabilities of surviving between the ages *x* and *x + 1*, *1px* |

IPUMS=Integrated Public Use Microdata Series; DHS= Demographic and Health Survey; WPP=World Population Prospects; NA=Not applicable. Equations, … (A); $m_{adrt}^{*}$ equals the expected net-migration for MWRA age *a* in division *d* region *r* at time *t*; $\beta_{0,adrt}$ is the random intercept term; $I_{1,adt}$is the random effects of the observations for each age *a,* in division *d* at time *t*; and $I_{2,drt}$ is the random effects of the observations for each division *d* in region *r* at time *t*; $\varepsilon_{adrt}$ is the residual of the hierarchical model. … (B); $f_{adrt}^{*}$ equals the expected ASFR for WRA age *a* in region *r* at time *t*; $\beta_{0,art}$ is the random intercept term, $z_{1,art}$is the random slope of the fertility observations for each age *a* and region *r* at time *t*; and $\varepsilon_{art}$ the residual of the hierarchical model.

## **Variables**

**Table S3: Definitions of family planning indicators**

| Family planning indicator | Definitions | |
| --- | --- | --- |
|  | **Numerator** | **Denominator** |
| Modern contraceptive prevalence | Currently married women aged 15–49 years using a modern method of contraception | Currently married women aged 15–49 years |
| Demand satisfied for family planning with modern methods | Currently married women aged 15–49 years with a demand for family planning, who are using a modern method of contraception | Currently married women aged 15–49 years, who have a demand for family planning |
| Unmet need for modern methods of family planning | Currently married women aged 15–49 years, who are not using a modern method of contraception and have a need for spacing or limiting | Currently married women aged 15–49 years |
| Met need for modern methods of family planning | Currently married women aged 15–49 years, who are using a modern method of contraception and have a need for spacing or limiting | Currently married women aged 15–49 years |

Married women include who are married or in a union. Total number of women with a demand for family planning (modern contraceptive prevalence + traditional contraceptive prevalence + unmet need). Modern contraceptive methods include sterilisations, oral contraceptive pills, intrauterine devices, injectables, implants, condoms, and lactational amenorrhea method, standard days method, emergency contraception and vaginal barrier methods [11].

# **Extended References**

1. QGIS Geographic Information System. Open Source Geospatial Foundation Project. [<http://qgis.org>]

2. Institut National de la Statistique (INS) et ICF International: Enquête Démographique et de Santé du Cameroun 2018. *Yaoundé, Cameroun et Rockville, Maryland, USA : INS et ICF [Producers] ICF [Distributor],* 2020.

3. Swanson DA, Tayman J: Subnational Population Estimates., vol. 31: Springer Science & Business Media.; 2012.

4. Smith SK: Tests of forecast accuracy and bias for county population projections. *Journal of the American Statistical Association* 1987, 82(400):991-991,012.

5. Alexander M, Alkema L: A Bayesian hierarchical model to estimate subnational populations of women of reproductive age. *Paper presented at PAA 2018* 2018.

6. Bryant JR, Graham PJ: Bayesian Demographic Accounts: Subnational Population Estimation Using Multiple Data Sources. *Bayesian Analysis* 2013, 8(3):International Society for Bayesian Analysis: 591–622.

7. Wheldon MC, Raftery AE, Clark SJ, Gerland P: Reconstructing Past Populations With Uncertainty From Fragmentary Data. *Journal of the American Statistical Association* 2013, 108(501):96-110.

8. Gelman A, Carlin BJ, Stern SH, Dunson BD, Vehtari A, Rubin BD: Bayesian Data Analysis Third Edition: Boca Raton. FL: CRC Press; 2020.

9. Chi G, Zhou X, Voss PR: Small-area population forecasting in an urban setting: a spatial regression approach. *Journal of Population Research* 2011, 28(2):185-201.

10. Gelman A, Rubin DB: Inference from Iterative Simulation Using Multiple Sequences. *Statist Sci* 1992, 7(4):457-472.

11. Ahmed S, Choi Y, Rimon JG, Alzouma S, Gichangi P, Guiella G, Kayembe P, Kibira SP, Makumbi F, OlaOlorun F *et al*: Trends in contraceptive prevalence rates in sub-Saharan Africa since the 2012 London Summit on Family Planning: results from repeated cross-sectional surveys. *The Lancet Global health* 2019, 7(7):e904-e911.
