# Supplementary material for: Contraceptive use, unmet need, and demand satisfied for family planning across Cameroon: a subnational study including indirect effects of COVID-19 and armed conflict on projections
Source: BMC Glob Public Health. 2024 Jul 3;2:40. doi: 10.1186/s44263-024-00071-4 (PMC11622886; doi:10.1186/s44263-024-00071-4)
Supplement: Supplementary file 2 — Additional file 2. Modelling Indirect Effects of Services Disruptions on Family Planning Projections. ⇒ Figure S1: Framework for the effects of health system components on coverage of health services. ⇒ Formula translating component reductions to overall change in family planning outcomes. ⇒ Table S4: Regression of components of services coverage against family planning indicators. [file 44263_2024_71_MOESM2_ESM.docx]

**Additional file 2**

**Modelling Indirect Effects of Services Disruptions on Family Planning Projections**


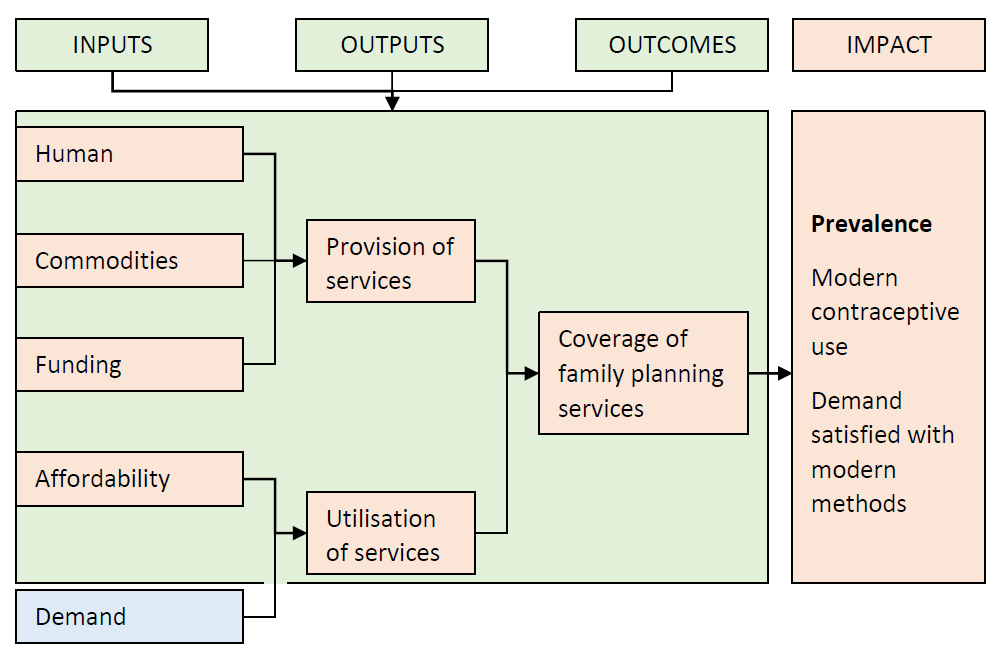


**Figure S1: Framework for the effects of health system components on coverage of health services**

Source: Adapted from the framework for the effects of health system components on coverage of health services [1].

**Formula translating component reductions to overall change in family planning outcomes**[1, 2]

$$Change in mFP indicator = \left[ \left( Provision \right)\left( 1 - x \right)\times\left( Utilisation \right)\left( 1 - y \right) \right]\ldots\ldots\ldots\ldots\ldots\ldots\ldots\ldots\ldots\ldots\ldots\ldots\ldots(i)$$

$$Change in mFP indicator = \left[ \left( HRH*FH*HB \right)\left( 1 - x \right) \right]\times\left[ \left( Demand*Affordability \right)\left( 1 - y \right) \right]\ldots\ldots\ldots(ii)$$

$$Change in mFP indicator = \left[ \left( HRH*FH*HB \right)\left( 1 - x \right) \right]\times\left[ \left( Demand*\left[ 1 - Poverty rate \right] \right)\left( 1 - y \right) \right]\ldots(iii)$$

Where; $mFP$=family planning indicator, and $x$and $y$=percentage reductions in provision and utilisation, respectively. Provision is composed of per capita, human resources, facilities, and budget for health, represented as $HRH$, $FH$, *and* $HB$, respectively. Demand is defined as the change in demand for family planning (the family planning estimation model (FPEM)estimate). The poverty rate equals the percentage of the population living below the poverty line. $x$ and $y$ equal the rates of reduction each of provision and utilization, respectively. FP indicators and demand are defined in terms of changes from 2020–2030 to eliminate the advantage for regions where levels are currently more favourable. Annual changes in FP indicator and demand, 2020–2030, were sourced as the median FPEM estimates. Observed changes in each FP indicator in region *r* was assumed to be normally distributed around the true change $F_{r,t}$ with some sampling error${s^{2}}_{r,t}$ . Changes in FP indicators for each region and year were captured following a Bayesian model which was built such that annual change, 2020–2030, will be influenced by corresponding changes in health services provision and utilisation as;

$$F_{r,t}^{*}=\beta_{0}+ \beta_{1,r,t} . P_{1,r,t}\left( 1-x \right)+ \beta_{2,r,t} . U_{2,r,t}\left( 1-y \right)+ R_{r,t}+ \varepsilon_{r,t}$$

Where; $F_{r,t}^{*}$ change in FP indicator; $\beta_{0}$ is the fixed intercept term, $\beta_{1,r,t}$ and $P_{1,r,t}$ equal the coefficient and observation associated with provision; $\beta_{2,r,t}$ and $U_{2,r,t}$ equal the coefficient and observation associated with utilisation, $x$ and $y$ are the terms for reductions in provision and utilisation, respectively, defined varyingly as 0%, 5%, 10%, and 25% per scenario; and $R_{2,r,t}$ equal the random effect between provision and utilisation; $\varepsilon_{r,t}$ is the error term; all for region *r* and time *t*.

Priors were specified as: $\beta_{0}\sim N(0, 100)$; $\beta_{1,r,t}\sim N(0, 100)$; $\beta_{2,r,t}\sim N(0, 100)$;

And $R_{r,t}\sim N(\left\{ F_{r,t}^{*}:\beta_{0} \right\}, {\sigma^{2}}_{r,t})$;${\sigma^{2}}_{r,t}\sim igamma(0.01, 0.01)$;

Estimates for changes in FP indicators for each region and year were computed as the median of samples from the posterior distributions via a Markov Chain Monte Carlo algorithm. Projections from 2021–2030 are the sum of the FPEM estimates in 2020, plus the model-derived changes for each year, from 2020–2029. Only the changes in mCPR were modelled as these showed statically significant associations with both provision and utilisation (Table S4).

**Table S4: Regression of components of services coverage against family planning indicators**

| **Regression coefficient** | | | | | |
| --- | --- | --- | --- | --- | --- |
| **Dependent variable** | **Independent variable** | | | | |
| Independent | Change in mCPR | Change in mUNFP | Change in mDSFP | Provision | Utilisation |
| Per capita human resources for health | 2.5×10^2^** | -1.8×10^2^* | 1.5×10^2^** | .. | .. |
| Per capita facilities for health | 1.1×10^3^* | -1.1×10^3^** | 7.6×10^2^** | .. | .. |
| Per capita health budget | 1.9×10^-4^ | -1.1×10^-4^ | -1.7×10^-5^ | .. | .. |
| Affordability | 7.2×10^-3^* | -6.0×10^-3^* | 4.2×10^-3^* | .. | .. |
| Change in demand for family planning | 7.9* | 1.7 | 1.4 | .. | .. |
| Provision | 3.9×10^2^* | -2.9×10^2^* | 1.7×10^2^ | .. | 2.9×10^4^* |
| Utilisation | 1.1×10^-2^** | -3.5×10^-3^ | 4.0×10^-3^ | 1.6×10^-5^* | .. |

**p* < 0.05; ***p* < 0.001; mCPR=modern contraceptive prevalence rate; mUNFP=unmet need for modern methods; mDSFP=demand satisfied in modern methods; ..=Not applicable

**Extended References**

1. Roberton T, Carter ED, Chou VB, Stegmuller AR, Jackson BD, Tam Y, Sawadogo-Lewis T, Walker N: Early estimates of the indirect effects of the COVID-19 pandemic on maternal and child mortality in low-income and middle-income countries: a modelling study. *The Lancet Global health* 2020, 8(7):e901-e908.

2. Nove A, Friberg IK, de Bernis L, McConville F, Moran AC, Najjemba M, Ten Hoope-Bender P, Tracy S, Homer CSE: Potential impact of midwives in preventing and reducing maternal and neonatal mortality and stillbirths: a Lives Saved Tool modelling study. *The Lancet Global health* 2021, 9(1):e24-e32.
