# Supplementary material for: Contraceptive use, unmet need, and demand satisfied for family planning across Cameroon: a subnational study including indirect effects of COVID-19 and armed conflict on projections
Source: BMC Glob Public Health. 2024 Jul 3;2:40. doi: 10.1186/s44263-024-00071-4 (PMC11622886; doi:10.1186/s44263-024-00071-4)
Supplement: Supplementary file 3 — Additional file 3. Supplementary Results. ⇒ Table S5: t-test results comparing the aggregates of subnational- to the national-level FPET estimates for Cameroon from 1990–2030. ⇒ Table S6: Counts of married women using, with unmet need, and demand satisfied for modern contraception across Cameroon’s regions and divisions in 2015 and projections for 2030. ⇒ Table S7: Projected levels of modern contraceptive use given varied percentage annual reductions in services coverage by regions of Cameroon, 2020–2030. [file 44263_2024_71_MOESM3_ESM.docx]

**Additional file 3**

**Supplementary Results**

**Table S5: *t*-test results comparing the aggregates of subnational- to the national-level FPET estimates for Cameroon from 1990–2030**

| **Family planning indicator** | **Aggregate level from the FPET** | **Mean** | **SE** | ***P*-value** |
| --- | --- | --- | --- | --- |
| Modern contraceptive prevalence | National | 13.35 | 0.84 |  |
|  | Regions | 13.97 | 0.90 | 0.31 |
|  | Divisions | 14.19 | 0.95 | 0.25 |
| Unmet need for modern methods | National | 31.14 | 0.41 |  |
|  | Regions | 31.07 | 0.44 | 0.55 |
|  | Divisions | 31.75 | 0.54 | 0.19 |
| Demand satisfied with modern methods | National | 29.24 | 1.62 |  |
|  | Regions | 28.23 | 1.67 | 0.67 |
|  | Divisions | 27.12 | 1.67 | 0.82 |

FPET=Family planning estimation tool; SE = Standard error; *p*>0.05 indicate a statistically insignificant difference between the estimates.

**Table S6: Counts of married women using, with unmet need, and demand satisfied for modern contraception across Cameroon’s regions and divisions in 2015 and projections for 2030**

| COUNTRY  Region  Division | Number of MWRA using modern contraception in 2015 | Number of MWRA using modern contraception in 2030 | Number of MWRA with unmet need for modern methods in 2015 | Number of MWRA with unmet need for modern methods in 2030 | Number of MWRA with demand satisfied with modern methods in 2015 | Number of MWRA with demand satisfied with modern methods in 2030 |
| --- | --- | --- | --- | --- | --- | --- |
| CAMEROON | **544984 (435405–671788)** | **1262440 (602527–2215287)** | **996054 (858929–1158367)** | **1494394 (1024856–2063763)** | **1174374 (968571–1391641)** | **2505675 (1459822–3584353)** |
| Adamawa | **15920 (12654–19781)** | **26260 (11198–56375)** | **50701 (43554–58822)** | **75224 (53277–102706)** | **44912 (36090–54326)** | **72649 (35199–125559)** |
| Djerem | 2057 (1607–2631) | 4171 (1981–7829) | 5563 (4733–6479) | 7387 (5284–9834) | 5719 (4577–6988) | 8837 (4869–13648) |
| Faro et Déo | 195 (142–272) | 24 (9–64) | 2806 (2328–3330) | 3097 (2106–4357) | 861 (614–1205) | 99 (34–288) |
| Mayo Banyo | 1185 (903–1534) | 1739 (734–3971) | 7476 (6176–8879) | 11734 (7965–16428) | 4945 (3783–6420) | 6447 (2740–13219) |
| Mbéré | 1207 (911–1589) | 2316 (991–5383) | 10389 (8827–12031) | 12174 (8574–16826) | 3824 (2882–5041) | 7700 (3318–15177) |
| Vina | 8383 (6507–10748) | 18319 (7917–36186) | 22920 (19350–26650) | 42822 (30566–57435) | 21716 (17319–26943) | 43523 (21645–70912) |
| Centre | **162866 (134750–194888)** | **436173 (228835–683197)** | **201628 (174544–231559)** | **326449 (211425–447912)** | **279383 (236417–321249)** | **736185 (477653–975238)** |
| Haute Sanaga | 2333 (1867–2882) | 3188 (1587–5646) | 5510 (4900–6193) | 4660 (3305–6153) | 3936 (3229–4726) | 5614 (3224–8500) |
| Lekié | 8560 (7004–10310) | 10775 (5803–16955) | 9968 (8648–11432) | 7444 (4820–10337) | 15476 (13188–17757) | 19227 (12784–25123) |
| Mbam et Inoubou | 6904 (5734–8220) | 10835 (6186–16119) | 6345 (5427–7376) | 5305 (3134–7808) | 11749 (10155–13309) | 18040 (12461–22443) |
| Mbam et Kim | 3319 (2668–4116) | 5223 (2854–8004) | 4490 (3848–5214) | 3629 (2384–5159) | 6304 (5234–7421) | 8735 (5754–11327) |
| Mefou et Afamba | 4968 (4106–5931) | 7356 (4152–11232) | 4442 (3795–5146) | 4888 (3145–6883) | 8297 (7196–9475) | 11823 (7915–15346) |
| Mefou et Akono | 1528 (1248–1829) | 1460 (786–2345) | 1656 (1424–1910) | 1016 (628–1463) | 2610 (2228–2987) | 2555 (1659–3405) |
| Mfoundi | 117614 (96098–139184) | 366614 (198308–568929) | 149781 (128204–172095) | 283446 (189196–394600) | 204903 (173888–236648) | 622612 (406697–819380) |
| Nyong et Kéllé | 4051 (3270–4966) | 5548 (2797–9524) | 8729 (7875–9636) | 8835 (6391–11263) | 5711 (4682–6871) | 8414 (4743–12814) |
| Nyong et Mfoumou | 2206 (1749–2764) | 3957 (1914–6906) | 4310 (3700–4964) | 5154 (3660–6792) | 4915 (4074–5914) | 7110 (4103–10451) |
| Nyong et So'o | 6695 (5512–7982) | 13339 (7334–19947) | 5400 (4583–6302) | 6447 (3706–9620) | 11367 (9891–12864) | 23115 (15703–28839) |
| East | **25951 (20645–32091)** | **65042 (36386–96942)** | **41935 (35238–49475)** | **40363 (24340–63299)** | **50653 (41070–60998)** | **103961 (67347–133947)** |
| Boumba et Ngoko | 3545 (2644–4454) | 16099 (11714–19605) | 8236 (6984–9547) | 3066 (1523–5468) | 6158 (4691–7559) | 20445 (16893–22582) |
| Haut Nyong | 3829 (3036–4823) | 9096 (5113–14112) | 6547 (5545–7530) | 4764 (2888–7010) | 8962 (7387–10642) | 16465 (11279–20634) |
| Kadey | 6294 (4945–7927) | 19149 (11514–27161) | 10798 (9040–12677) | 10384 (6272–16232) | 13410 (10917–16041) | 28898 (19925–36048) |
| Lom et Djerem | 9896 (7635–12472) | 25484 (14259–39726) | 15821 (13248–19098) | 18168 (11608–28324) | 19907 (15650–23980) | 43413 (27884–56969) |
| Far North | **31670 (25087–40376)** | **90869 (40553–189312)** | **143980 (121741–169813)** | **194692 (133682–272575)** | **112477 (88504–141571)** | **266870 (138673–438565)** |
| Diamaré | 11181 (8795–14180) | 22489 (10282–44127) | 34817 (29260–40591) | 39434 (27787–54636) | 30917 (24599–38181) | 60058 (32700–92553) |
| Logone et Chari | 5907 (4561–7677) | 30521 (13446–60615) | 27714 (23281–32839) | 45766 (31047–64675) | 21315 (16335–27251) | 81874 (43204–125165) |
| Mayo Danay | 2784 (2116–3617) | 7836 (3410–16667) | 19674 (16257–23424) | 23142 (15654–33159) | 11968 (8877–15825) | 28539 (13780–50102) |
| Mayo Kani | 2027 (1549–2647) | 6581 (2874–14189) | 15706 (13115–18613) | 17461 (11918–24162) | 7776 (5849–10173) | 21436 (10473–36899) |
| Mayo Sava | 1841 (1416–2371) | 3277 (1404–7234) | 12639 (10630–15106) | 11693 (7872–16535) | 6933 (5234–8919) | 11866 (5527–22021) |
| Mayo Tsanaga | 6738 (5196–8845) | 35703 (16183–69388) | 28450 (23516–34073) | 51388 (35336–72293) | 29946 (23121–38160) | 91632 (48768–139298) |
| Littoral | **111786 (88020–137301)** | **265250 (135478–442240)** | **186646 (158353–217344)** | **287639 (198679–404877)** | **198648 (159585–238231)** | **477114 (287753–665708)** |
| Moungo | 6936 (5250–8812) | 11820 (6063–19419) | 18471 (16128–21034) | 14463 (10273–19443) | 12392 (9503–15263) | 19292 (11390–27522) |
| Nkam | 866 (688–1062) | 1203 (655–1853) | 1517 (1319–1720) | 878 (573–1206) | 1435 (1177–1713) | 1939 (1269–2556) |
| Sanaga Maritime | 3870 (3063–4826) | 3977 (1833–7487) | 7245 (6318–8293) | 7968 (5736–10463) | 7455 (6074–8855) | 7612 (3973–12276) |
| Wouri | 101076 (80278–123149) | 244729 (125232–410619) | 150807 (128085–177242) | 262580 (179165–366229) | 184375 (147823–218031) | 448380 (271344–626084) |
| North | **37949 (29973–47640)** | **94648 (42974–197195)** | **115748 (97367–134832)** | **194186 (136545–268733)** | **114462 (92181–139362)** | **285710 (148664–462251)** |
| Bénoué | 21518 (16878–27604) | 82197 (37462–160653) | 70675 (59798–82502) | 139382 (96826–192003) | 61754 (48835–76635) | 212885 (117052–329649) |
| Faro | 420 (326–540) | 328 (138–726) | 2517 (2125–2968) | 1407 (937–2004) | 1360 (1042–1748) | 1274 (565–2456) |
| Mayo Louti | 4098 (3156–5247) | 13314 (5782–29764) | 22533 (18812–26866) | 31836 (21631–46339) | 16314 (12360–20880) | 49121 (23892–84317) |
| Mayo Rey | 2616 (1985–3410) | 12662 (5578–26141) | 20894 (17466–24685) | 25948 (17724–36587) | 9190 (6959–12385) | 40607 (20689–65727) |
| Northwest | **50975 (41798–61459)** | **83119 (44289–134148)** | **60134 (51377–69891)** | **70034 (47079–96785)** | **92668 (78122–107585)** | **148726 (97146–200295)** |
| Boyo | 23 (11–47) | 99 (21–382) | 5649 (4579–6866) | 6068 (4131–8541) | 63 (32–125) | 246 (58–828) |
| Bui | 7736 (6165–9540) | 13672 (7126–22969) | 8885 (7528–10446) | 10530 (6940–14779) | 16799 (14105–19469) | 27735 (17822–37109) |
| Donga Mantung | 3175 (2487–3988) | 9745 (4776–16846) | 9634 (8169–11137) | 9516 (6449–13031) | 7985 (6389–9892) | 19013 (11522–26735) |
| Menchum | 28 (15–53) | 86 (23–314) | 6279 (4938–7856) | 6983 (4616–10045) | 98 (49–188) | 303 (77–1128) |
| Mezam | 20356 (16563–24309) | 44870 (26229–66110) | 17219 (13972–21228) | 26087 (16545–39022) | 33851 (28357–39040) | 72918 (49362–91610) |
| Momo | 1217 (743–1897) | 1935 (681–4534) | 5302 (4243–6480) | 4733 (3279–6435) | 3107 (1989–4537) | 4325 (1823–7770) |
| Ngo Ketunjia | 5223 (4285–6230) | 5993 (3128–9608) | 5412 (4654–6228) | 5459 (3488–7493) | 8460 (7209–9683) | 9544 (5866–13323) |
| West | **50565 (40237–62123)** | **79181 (39922–133115)** | **79865 (66740–94316)** | **85722 (58522–121541)** | **96911 (78770–115661)** | **146718 (88485–203161)** |
| Bamboutos | 11173 (8962–13727) | 16647 (9505–24588) | 13236 (11018–15743) | 11604 (7345–16685) | 18168 (14791–21533) | 25361 (16509–32859) |
| Haut Nkam | 3436 (2613–4385) | 3800 (1893–6478) | 5956 (4860–7256) | 5425 (3759–7437) | 6122 (4610–7605) | 6591 (3745–9726) |
| Hauts Plateaux | 100 (69–144) | 12 (4–35) | 2504 (2155–2878) | 1234 (854–1703) | 267 (184–384) | 45 (14–136) |
| Koung Khi | 933 (717–1183) | 666 (314–1197) | 1877 (1572–2186) | 959 (669–1335) | 1723 (1341–2125) | 1274 (718–1898) |
| Menoua | 8371 (6625–10274) | 9919 (5444–15136) | 10474 (8651–12706) | 7848 (5178–11262) | 14171 (11328–16934) | 16052 (10338–21212) |
| Mifi | 12969 (10053–16034) | 32212 (17874–49706) | 19580 (16396–23064) | 23511 (15365–34182) | 21842 (17240–26370) | 52689 (34739–69264) |
| Ndé | 1723 (1296–2216) | 4051 (2243–6155) | 3018 (2466–3687) | 2437 (1516–3519) | 3897 (2943–4771) | 6749 (4509–8631) |
| Noun | 8494 (6240–11244) | 21641 (10442–39025) | 21384 (17975–25524) | 27624 (19075–39262) | 23693 (18116–29708) | 47098 (27469–67448) |
| South | **25105 (20635–30259)** | **40569 (19521–74982)** | **36182 (31412–41098)** | **59262 (42458–79782)** | **47452 (40623–54737)** | **80376 (45747–121182)** |
| Dja et Lobo | 3781 (2977–4767) | 4033 (1810–8168) | 8314 (7138–9666) | 10069 (7039–13488) | 8601 (7014–10334) | 9751 (4926–16480) |
| Mvila | 8490 (6911–10376) | 21570 (11132–35353) | 11093 (9583–12774) | 21089 (13565–28974) | 15527 (13112–18063) | 35361 (21196–50085) |
| Océan | 5084 (3888–6493) | 9816 (4325–20011) | 16151 (14092–18257) | 30881 (23287–39570) | 9143 (7127–11412) | 18248 (9078–31773) |
| Vallée du Ntem | 3283 (2662–4027) | 6698 (3575–10477) | 3762 (3202–4388) | 4803 (3041–6781) | 6784 (5792–7836) | 11305 (7363–14926) |
| Southwest | **43297 (34562–53004)** | **69332 (35398–117189)** | **61630 (52933–72148)** | **74575 (51498–102743)** | **79722 (65357–93641)** | **126714 (78771–178324)** |
| Fako | 16626 (13174–20600) | 30492 (15331–53544) | 25349 (21403–29856) | 39800 (28443–53932) | 32761 (26588–38954) | 57523 (34202–83907) |
| Koupé Manengouba | 3512 (2364–4758) | 4701 (2648–6598) | 4280 (3248–5731) | 1719 (847–2988) | 5456 (3732–7062) | 6713 (4560–8141) |
| Lebialem | 2671 (1668–3918) | 5990 (2612–10105) | 4644 (3539–6184) | 2854 (1567–5053) | 5640 (3670–7559) | 11190 (6648–14240) |
| Manyu | 8336 (5876–11050) | 17731 (9355–26293) | 8827 (6984–11065) | 6558 (3168–11615) | 13192 (9891–16325) | 27187 (17690–33239) |
| Meme | 10627 (8487–12994) | 19686 (10559–30726) | 12261 (10402–14537) | 11945 (7525–17270) | 20317 (16880–23504) | 35590 (24090–45535) |
| Ndian | 2532 (1631–3615) | 3446 (1558–5746) | 4263 (3238–5516) | 2881 (1654–4694) | 4493 (2972–6095) | 5422 (2816–7684) |

Estimates are in actual counts (95% Credible Interval); MWRA=married or in-union women of reproductive age; Modern methods refer to modern contraceptive methods. Slight discrepancies between higher vs combined lower administrative unit estimates stem from differences in the number of observations and data points available per family planning indicator and separate runs of the Family Planning Estimation Model for each administrative level.

**Table S7: Projected levels of modern contraceptive use given varied percentage annual reductions in services coverage by regions of Cameroon, 2020–2030**

| Scenario | | Change in modern contraceptive prevalence rate, 2020–2030 (%) | | | | | | | | | | |
| --- | --- | --- | --- | --- | --- | --- | --- | --- | --- | --- | --- | --- |
|  | | **Adamawa** | **Centre** | **East** | **Far North** | **Littoral** | **North** | **Northwest** | **West** | **South** | **Southwest** | **Cameroon** |
| FPEM estimate | | 2.9 | 7.6 | 8.5 | 3.4 | 7.5 | 3.4 | 7.0 | 7.2 | 5.0 | 6.5 | 6.6 |
| Reduction in coverage of services | |  |  |  |  |  |  |  |  |  |  |  |
| Provision | Utilisation |  |  |  |  |  |  |  |  |  |  |  |
| None | None | 4.6 | 7.4 | 11.4 | 1.2 | 7.7 | 4.6 | 6.2 | 3.1 | 6.0 | 9.5 | 4.7 |
| None | Small | 4.7 | 11.2 | 14.2 | 1.1 | 9.0 | 4.4 | 6.4 | 7.1 | 6.9 | 13.3 | 6.2 |
| None | Moderate | 4.7 | 14.1 | 16.3 | 1.0 | 9.9 | 4.3 | 6.5 | 10.1 | 7.6 | 16.7 | 7.3 |
| None | Large | 4.7 | 19.3 | 20.2 | 0.8 | 11.6 | 4.1 | 6.8 | 15.5 | 8.9 | 23.0 | 9.3 |
| Small | None | 3.4 | 2.1 | 9.4 | 0.8 | 6.0 | 4.2 | 4.7 | 0.0 | 1.4 | 6.4 | 3.2 |
| Small | Small | 3.4 | 4.8 | 11.2 | 0.7 | 6.7 | 4.0 | 4.8 | 2.7 | 1.9 | 8.8 | 4.0 |
| Small | Moderate | 3.4 | 6.8 | 12.6 | 0.6 | 7.3 | 3.9 | 4.8 | 4.8 | 2.4 | 11.8 | 4.7 |
| Small | Large | 3.3 | 10.6 | 15.3 | 0.4 | 8.3 | 3.6 | 4.9 | 8.7 | 3.3 | 16.3 | 6.0 |
| Moderate | None | 2.5 | -1.9 | 7.9 | 0.6 | 4.6 | 3.9 | 3.6 | -2.4 | -2.2 | 4.6 | 2.0 |
| Moderate | Small | 2.4 | -0.1 | 9.0 | 0.4 | 5.0 | 3.7 | 3.6 | -0.6 | -1.8 | 6.6 | 2.4 |
| Moderate | Moderate | 2.4 | 1.3 | 9.9 | 0.3 | 5.3 | 3.5 | 3.5 | 0.8 | -1.5 | 8.1 | 2.8 |
| Moderate | Large | 2.3 | 4.0 | 11.7 | 0.0 | 5.9 | 3.2 | 3.5 | 3.6 | -0.9 | 11.3 | 3.5 |
| Large | None | 0.8 | -9.1 | 5.0 | 0.0 | 2.2 | 3.4 | 1.6 | -6.7 | -8.7 | 1.2 | -0.1 |
| Large | Small | 0.7 | -8.8 | 5.0 | -0.1 | 1.9 | 3.1 | 1.4 | -6.4 | -8.7 | 1.5 | -0.4 |
| Large | Moderate | 0.5 | -8.5 | 5.0 | -0.3 | 1.8 | 3.0 | 1.2 | -6.2 | -8.7 | 1.7 | -0.6 |
| Large | Large | 0.3 | -7.8 | 5.7 | -1.0 | 1.2 | 2.4 | 0.3 | -5.7 | -9.2 | 3.0 | -1.5 |

FPEM=Family planning estimation model; Estimates also reflect existing inequalities in the provision of health services coverage. The Centre, West, and South are three regions with substantially higher health services provision per capita. None = 0%, Small = -5%, Moderate = -10%, and Large = -25% annual reduction in coverage of services (i.e., either ‘Provision’ or “Utilisation)
